# Comparison of four commercial solid-phase micro-extraction (SPME) fibres for the headspace characterisation and profiling of gunshot exhausts in spent cartridge casings

**DOI:** 10.1007/s00216-022-04129-w

**Published:** 2022-05-24

**Authors:** Matteo D. Gallidabino, Kelsey Bylenga, Stephanie Elliott, Rachel C. Irlam, Céline Weyermann

**Affiliations:** 1 Centre for Forensic Science, Department of Applied Sciences, Northumbria University Newcastle, Newcastle upon Tyne, NE1 8ST UK; 2 King’s Forensics, Department of Analytical, Environmental & Forensic Sciences, King’s College London, 150 Stamford Street, London, SE1 9NH UK; 3 National Forensic Laboratory Services, Royal Canadian Mounted Police, 14200 Green Timbers Way, Surrey, V3T 6P3 Canada; 4 School of Natural and Environmental Sciences, Newcastle University, Newcastle upon Tyne, NE1 7RU UK; 5 Ecole des Sciences Criminelles, Faculté de Droit, des Sciences Criminelles et d’Administration Publique, Université de Lausanne, 1015 Lausanne-Dorigny, Switzerland

**Keywords:** Firearms, Ammunition, Sampling, Gunshot residue, Volatile compounds, Gas chromatography

## Abstract

Headspace solid-phase micro-extraction (SPME) is a promising technique for the characterisation and profiling of gunshot exhausts in spent cartridge casings, especially for health and environmental risk assessments, as well as forensic purposes. To date, however, no comprehensive investigation has been carried out to objectively assess the kinds of compound released during a discharge that can be recovered by this approach, the selectivity of the main commercially available fibres, and their relative performances for the analysis of gunshot exhausts and the discrimination of different ammunition types. This study aimed to fill this gap. Gunshot exhausts in spent cartridge casings from four different ammunition types were analysed by GC–MS, after extraction with four different commercial fibres: 100 μm polydimethylsiloxane (PDMS), 85 μm polyacrylate (PA), 65 μm polydimethylsiloxane/divinylbenzene (DVB), and 85 μm carboxen/polydimethylsiloxane (CAR). Results showed that, overall, a total of 120 analytes could be observed across the cartridges, but the different tested fibres also displayed distinct performances, which were, to some extent, complementary for the characterisation of gunshot exhausts. DVB, in particular, recovered the most compounds simultaneously. On the other hand, the observed variability between measurements was also high, making it a poor candidate for (semi-)quantitative applications (e.g. estimation of time since discharge and/or source profiling). In this regard, PA demonstrated the highest potential for broad use and implementation in multi-purpose methods.

**Supplementary Information:**

The online version contains supplementary material available at 10.1007/s00216-022-04129-w.

## Introduction

According to a recent survey, there are more than one billion firearms in the world and these can be used for several different purposes, spanning legal activities (e.g. sport shooting or policing) to criminal and military operations [1]. As a consequence, they have a large impact on society, and the analysis and characterisation of their exhausts from spent cartridge casings are important for a number of purposes. These include assessing the risks to human health or the environment of the compounds released [2, 3] and providing useful information in criminal investigations [4, 5]. In forensic science, in particular, the analysis of gunshot exhausts from spent cartridge casings has been proposed for estimating the time since discharge and/or establishing chemical links with a specific bullet entry hole [6, 7].

Gunshot exhausts (also known as gunshot residues, or GSRs, in a forensic context) are heterogeneous mixtures of different materials that include species of various natures and/or origin. These can generally be classified into three main categories according to their formation mechanism: (i) primary discharge products (e.g. carbon dioxide and nitrogen oxides), (ii) pyrolytic discharge by-products (e.g. benzonitrile and naphthalene), and (iii) residual ammunition compounds (e.g. ethyl centralite and dibutyl phthalate) [7–9]. Gas chromatography–mass spectrometry (GC–MS) has proven suitable for the analysis of all these kinds of analytes directly from spent cartridge casings, but a main problem in this specific context is the sampling. For this, numerous techniques have been suggested, including dynamic or static headspace extraction using evacuated steel cylinders, sorptive traps (containing Tenax, XAD resin or activate charcoal) and, more recently, headspace sorptive extraction (HSSE) [10–15]. Whilst all these techniques allow the simultaneous and efficient recovery of a large range of chemical species, they also typically require relatively complex setups that, for some of them, are expensive, invasive, time consuming and not easily automatable (e.g. HSSE), characteristics that strongly limit their potential application in routine analysis.

Solid-phase micro-extraction (SPME) is a solvent-free sampling technique that involves a miniaturised device comprising a small fused silica fibre coated with a thin layer of sorbent phase [16]. Thanks to its design, it offers an efficient solution to the aforementioned limitations. Andrasko and co-workers first proposed SPME for the analysis of gunshot exhausts directly from spent cartridge casings to estimate the time since discharge [17–21]. Results showed good uptakes for a selection of target compounds, through the use of a fibre coated with polyacrylate or carboxen/polydimethylsiloxane. More recent works presented further improvements by conditioning the samples in closed environments and/or using different fibre types, such as polydimethylsiloxane and polydimethylsiloxane/divinylbenzene [22–26]. Whilst promising results were reported, all published works using SPME mainly focused on the targeted analysis of a few selected compounds with similar chemistries and, often, with minimal method optimisation. In particular, no comprehensive investigation has been carried out to date to objectively assess the kinds of compound released during a discharge that can be recovered by headspace SPME, the selectivity of the main commercially available fibres and their relative performances for the characterisation and profiling of gunshot exhausts.

Therefore, the aim of this work was to address this gap through an empirical study, in a similar way to that which has previously been done in other fields where SPME has been suggested [27–31], in order to support the development of future methods. For this purpose, gunshot exhausts in spent cartridge casings from four ammunition types were analysed using different SPME fibres: 100 μm polydimethylsiloxane (PDMS), 85 μm polyacrylate (PA), 65 μm polydimethylsiloxane/divinylbenzene (DVB) and 85 μm carboxen/polydimethylsiloxane (CAR). Extracted compounds were first identified via non-targeted analysis. Then, their signals were compared. Different extraction temperatures (20 °C and 80 °C) were also used, in order to take into account the effect of the extraction conditions.

## Experimental

### Chemicals and materials

Reference standards of 57 compounds were purchased from different suppliers; the complete list is provided in Table S1 in the Electronic supplementary materials (ESM). For each substance, a stock solution was prepared at a concentration of 1 mg·mL^−1^ in methanol (puriss. grade) purchased from Sigma/Aldrich (Buchs, Switzerland), except for anthracene, chrysene and benzo[a]pyrene, which were dissolved in chloroform (purum grade), also purchased from Sigma/Aldrich.

Four different ammunition types of .357 Magnum calibre were purchased in 2011 from suppliers in Switzerland (see Table 1). These were produced by either Geco (Fürth, Germany), Sellier & Bellot (Vlašim, Czech Republic), Samson (Rammat-Hasharon, Israel) or Magtech (Lino Lakes, Minnesota). From each ammunition box, two cartridges were opened using a kinetic hammer and the smokeless powder was analysed by GC–MS using a method adapted from Romolo [32] (see results in Table 1). Previous works showed that there was no particular difference in the amounts or the chemical profiles of gunshot exhausts released by revolver or pistol calibres [11]. As a consequence, cartridges belonging to the .357 Magnum calibre were chosen here primarily because they could fit easily through the opening of the headspace vials used.

**Table 1:** Cartridges selected for the study, their characteristics, and the composition of their smokeless powders determined by GC–MS. “M” indicates a major compound (> 1% of the total area count) and “m” a minor compound (0.2–1% of the total area count); “FMJ” indicates full metal jacket bullets and “JSP” jacket soft point bullets. Masses are given in grains (1 grain = 64.80 mg)

*Abbreviation*	*Characteristics*					*Smokeless powder composition*						
	*Calibre*	*Brand*	*Bullet mass [gr]*	*Bullet type*	*Powder weight [gr]*	*NG*	*DPA*	*EC*	*DBP*	*2NDPA*	*AKII*	*DF*
Ge357	*.357 Magnum*	*Geco*	158	FMJ	6.3	M	M	M	m	–	M	–
Se357	*.357 Magnum*	*Sellier & Bellot*	158	FMJ	6.2	M	M	–	M	–	–	–
Sa357	*.357 Magnum*	*Samson*	125	JSP	17.2	M	M	M	M	m	–	m
Ma357	*.357 Magnum*	*Magtech*	158	FMJ	14.6	M	M	M	M	–	–	–

*NG* nitroglycerin, *DPA* diphenylamine, *EC* ethylcentralite, *DBP* dibutylphthalate, *2NDPA* 2-nitrodiphenylamine, *AKII* 1-methyl-3,3-diphenylurea (akardite II), *DF* dioctyl fumerate

### Test shooting

Test shootings were carried out using a Colt Python revolver chambered for .357 Magnum cartridges. Before starting the test shootings, the firearm was carefully cleaned with dry cleaning patches. All test cartridges then were fired, in a completely random order, by loading them singly in the cylinder. Cartridges were handled whilst wearing latex gloves, in order to minimise contamination from hands. Latex gloves were preferred over nitrile, since preliminary tests showed that the latter introduced several unwanted impurities into samples, especially long-chain aliphatic compounds. Weapons were never greased between test shootings. The exact same methodology was adopted in a previous study by Gallidabino et al. [11], and it proved to be reliable and (cross-)contamination free.

Spent casings were immediately recovered after discharge and put in 10-mL headspace-dedicated screw glass vials (Supelco, Buchs, Switzerland), which were immediately closed with 18-mm magnetic screw caps containing 1.8-mm polytetrafluoroethylene/silicon septa (Supelco). The smallest vials were chosen, in order to maximise headspace concentrations of volatile compounds. Glassware was conditioned in a ventilated laboratory oven at 80 °C for 8 h before use. The spent casings in the vials were left to equilibrate overnight, before being inserted in the GC tray for automated SPME analysis.

### Headspace SPME of spent casings

Automated headspace SPME analyses were performed using a Gerstel Multi-purpose sampler MPS2-Twister (Gerstel, Sursee, Switzerland), mounted on an Agilent 6890 N gas chromatograph coupled to an Agilent 5973 mass selective detector (Agilent Technologies, Basel, Switzerland). Four fibres with different coatings were evaluated: 100 μm polydimethylsiloxane (PDMS), 85 μm polyacrylate (PA), 65 μm polydimethylsiloxane/divinylbenzene (DVB) and 85 μm carboxen/polydimethylsiloxane (CAR). All fibres were purchased from Supelco, and each was conditioned according to the manufacturer’s instructions, before the start of each analytical sequence. PDMS and DVB were conditioned at 250 °C for 30 min, PA was conditioned at 280 °C for 60 min and CAR was conditioned at 300 °C for 60 min. During the analyses, the fibres were also briefly re-conditioned at 280 °C (adjusted to 270 °C for the DVB fibre) for 1 min before each extraction and for 15 min after injection and desorption had finished.

Extractions were carried out by piercing the cap septa with the SPME needles, so the fibres could be suspended in the middle of the spent casings without touching their inner walls. The vial penetration depth was set to 38 mm. Previous tests indicated that, compared to 20 °C, a sampling temperature of 80 °C promoted better extraction of less volatile compounds, but the recovery of the most volatile compounds decreased in some cases [11]. Different samples of the same ammunition type (same box) were thus analysed at the two temperatures, in order to guarantee the detection and identification of a larger range of compounds. For extraction at 80 °C, the spent casings were automatically incubated for 7 min in the MPS2 agitator/incubator to allow headspace equilibration. Spent casings extracted at 20 °C were extracted directly from the GC tray and not incubated, as this matched the room temperature around the GC–MS instrument (air-conditioned laboratory).

Preliminary tests showed that, for all the tested fibres, an extraction time of 40 min was a reasonable compromise to reach equilibration for most compounds in gunshot exhausts, without important displacement effects on the two mixed-phase ones (DVB and CAR). These observations are in line with published data (e.g. [33]). Hence, an extraction time of 40 min was used in all experiments. For each ammunition type, three different cartridges from the same box were analysed per temperature and fibre type, for a total of 96 cartridges spent. Procedural blanks were also systematically carried out. These consisted of empty vials that had been handled in the same way as those used for sampling the spent casings and had been in contact with the same environments. Three blanks per extraction condition were analysed.

### GC–MS analysis

After automated headspace SPME, fibres were desorbed in the GC injector for 2 min in splitless mode. The injector temperature was maintained at 280 °C for all fibres, with the exception of DVB for which it was set at 270 °C. No solvent delay was used.

GC separation was performed on a DB-1MS (30 m × 0.25 mm × 0.25 μm) column from Agilent. The carrier gas was helium and column flow was maintained at 1.2 mL·min^−1^. The oven ramp was programmed as follows: 40 °C for 2 min, increased to 100 °C at 10 °C·min^−1^, increased to 200 °C at 5 °C·min^−1^, increased to 280 °C at 10 °C·min^−1^ and held at this temperature for 10 min (total chromatographic time: 46 min). The transfer line between the column and the MS was held at 280 °C. Ionisation was carried out through electron impact (EI). Masses were scanned from *m/z* 40 to 500. MS source and quadrupole temperatures were 230 °C and 150 °C, respectively.

### Injection of standard solutions and quality control

Preliminary tests showed that direct liquid injection of reference standard solutions caused a shift in retention times compared to SPME injections, likely due to a faster mass transfer from the injector to the column. In order to allow retention time comparison, therefore, reference standard solutions were also subject to headspace SPME. For each substance, a working solution was obtained by further tenfold dilution of the respective stock solution to a concentration of 0.1 mg·mL^−1^ using deionised water. One millilitre of this solution was then analysed in a similar way to the spent casings, i.e. by placing it in a 10-mL headspace-dedicated screw-capped glass vial (Supelco) closed with an 18-mm magnetic screw cap containing a 1.8-mm polytetrafluoroethylene/silicon septum (Supelco). Standard compounds were sampled using a PDMS or PA fibre, depending on their chemistry (see Table S1). Vial penetration depth was set to 21 mm.

All working solutions could be extracted at 120 °C for 40 min. Exceptions were 2,4-dinitrodiphenylamine, 4-nitrodiphenylamine, 2,4-dinitrotoluene and 2,6-dinitrotoluene, which decompose at this temperature. For these substances, an extraction temperature of 80 °C was used instead. Split mode was used and set at 5:1. As preliminary tests showed the possibility for some fibres to pick up some residual solvents from the preparation of the stock solutions (i.e. methanol and chloroform), a solvent delay of 4 min was applied to safeguard the MS filament. This was adjusted to 3.5 min and 2.4 min for the analyses of the working solutions of toluene and benzene, respectively, due to their elution before 4 min. The same GC separation settings used for SPME analyses were applied. Quality control samples were also included in the analyses, in order to monitor the GC–MS response and guarantee data comparability. For this purpose, naphthalene was used, as it was efficiently extracted by all fibre coatings. A fresh working solution of naphthalene at 0.1 mg·mL^−1^ was, therefore, prepared before each run and 1 mL analysed after each three spent casings. All extractions were carried out at 20 °C for 40 min.

### Peak labelling and initial data processing

Chromatograms were initially processed using the ChemStation software provided by Agilent. The same non-targeted workflow reported in Gallidabino et al. [11] was used for peak detection and labelling. Briefly, chromatograms were automatically analysed by the AMDIS sub-routine (NIST, Gaithersburg, Maryland) integrated in ChemStation, in order to perform peak picking and spectral deconvolution. This allowed a list of probable compounds to be obtained, which were preliminarily identified through the comparison of their mass spectra with the NIST08 library (NIST). As AMDIS sometimes returns high match indices, hits were further evaluated by visual comparison and, eventually, manually rectified. Standard solutions were injected to confirm the identity of some compounds of particular interest. Detection of a compound was accepted if its mass fragmentation pattern was consistent with the results of the library search, and for the molecules for which standards were available, retention time had to also match (< 0.5%) [34].

After labelling, target ions were selected for all compounds (Table S2) and the corresponding peak areas were extracted. The presence of a compound in a particular ammunition type was accepted if, in at least one of the analysed cartridges, the intensity of its target ion was greater than the average signal in the procedural blanks plus three times the standard deviation [35].

For the following performance assessment (see below), peak areas were extracted for those compounds detected in gunshot exhausts with a reference standard (*n* = 50 in total) from the extracted-ion chromatogram (EIC) corresponding to the *m/z* value of their respective target ion. This allowed “chemical profiles”, composed of 50 features (compounds) each, to be built for each experiment performed. However, only a subset of 14 analytes of particular interest (10 explosion by-products and 4 residual ammunition components) was used for visualisation purposes and statistical resampling procedures, namely benzaldehyde, benzonitrile, m-tolunitrile, naphthalene, 2-methylnaphthalene, biphenyl, acenaphtylene, 1-naphthalenecarbonitrile, phenanthrene, pyrene, 2-ethyl-1-hexanol, diphenylamine, ethyl centralite and dibutyl phthalate. These were chosen based on published literature, as they cover the main classes of compounds in gunshot exhausts and typically represent the most concentrated [10–13]. Therefore, they are likely best for informing the selection of the best conditions for different applications.

### Assessment of the extraction performances

For each set of experiments, peak areas extracted in EICs from the three corresponding replicate analyses were initially averaged. The coefficient of variation (CV) was also determined as follows:

1 $$\mathrm{CV}=\frac{s}{\overline{A} }$$

where $\overline{A }$ is the average peak area determined from the three analyses for a given compound of interest and *s* is the respective standard deviation. Average peak areas and CVs were used to assess the recovery efficiencies and reproducibility, respectively, of the tested fibres within a specific ammunition type.

For a more efficient analysis and comparison of the recovery efficiencies, a relative concentration factor (RCF) was also calculated following a strategy inspired by Bicchi et al. [27]. The RCF has been defined as follows:

2 $$\mathrm{RCF}=\frac{\overline{A} }{{\overline{A} }_{\mathrm{MAX}}}$$

where ${\overline{A} }_{\mathrm{MAX}}$ is the largest of the average peak area values observed for the specific compound of interest across the other fibre and temperature conditions. The RCF can, therefore, assume a value from 0 to 1. A value close to 1 indicates a very high recovery efficiency at the considered conditions and vice versa.

Both CVs and RCFs were initially calculated for each target compound after the analysis of each ammunition type. Between-ammunition values were also calculated by averaging the four corresponding values determined on the four ammunition types. These were used for a more objective and ammunition-independent evaluation of the recovery efficiencies and reproducibility.

### Assessment of the source-discrimination performances

To better assess the ability of the tested fibres to discriminate gunshot exhausts according to their source (i.e. ammunition type), chemical profiles were further analysed by principal component analysis (PCA).

There is no agreement in the literature regarding the most discriminant compounds for source profiling of gunshot exhausts. Therefore, only the aforementioned selection of 14 analytes of particular interest was considered for this investigation, in order to avoid including uninformative features in the analysis and bias results. A statistical resampling method was also used, in order to further study the variability introduced by different selections of the 14 analytes and provide a clearer overall picture. This involved the application of the following pipeline: (1) selection of a subset of analytes from the original pool of 14, (2) extraction of the related peak areas across samples, (3) removal of analytes with near-zero variance (relevant only for data collected using the CAR fibre), (4) normalisation of the peak areas by their total sum (profile by profile), (5) further normalisation by mean-centering and scaling (analyte by analyte), (6) application of PCA to the pre-treated dataset and (7) calculation of the degree of separation between the source-related clusters. For the first step (i.e. selection of a subset of analytes), all the different combinations between 8 and 14 analytes were tested, for a total of 6476 subsets for each extraction condition.

To objectively assess the degree of cluster separation on the PCA score plots, the *J*_2_ criterion was used, as described in Anderson et al. [36]. This involved determining the *J*_2_ index for each PCA score plot, according to the following equation:

3 $${J}_{2}=\frac{\left|{S}_{\mathrm{w}}+{S}_{\mathrm{b}}\right|}{\left|{S}_{\mathrm{w}}\right|}$$

where *S*_w_ is the within-source scatter and *S*_b_ is the between-source scatter. A large value of *J*_2_ indicates well-separated and tightly clustered groups of analyses and vice versa. Only the first two principal components were considered for this analysis.

### Software

R statistical computing software v. 4.0.2 was used for most data treatment. In particular, the following packages were adopted: “car” for analysis of variance (ANOVA), “VennDiagram” for obtaining the Venn diagrams, “stats” for principal component analysis (PCA) and “ggplot2” for plotting the heatmaps.

## Results and discussion

### Compounds detected

**Fig. 1:**
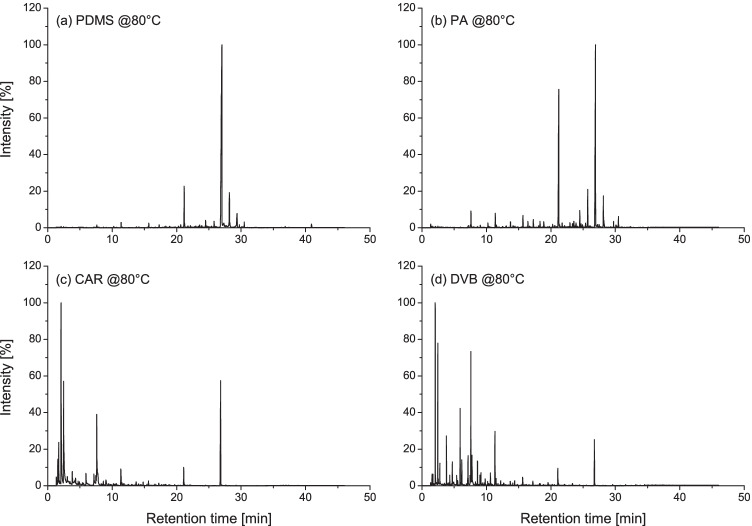
Examples of total ion chromatograms (TICs) obtained after headspace SPME extraction at 80 °C of different spent cartridge casings belonging to the same ammunition type (Ma357), using each of the four fibres tested in this work. Differences between the chemical profiles can be clearly visualised

Spent cartridge casings from four ammunition types were extracted by headspace SPME after discharge using four different fibres and at two different extraction temperatures. In general, the obtained total ion chromatograms (TICs) were characterised by a large number of peaks (Fig. 1). Their identities were investigated by non-targeted analysis, and a total of 120 analytes were observed to be recurrent between TICs. Amongst them, 113 could be labelled; the full list is reported in Table S2.

Compounds identified varied greatly from a chemical point of view and covered a broad range of volatilities and polarities. In particular, they could be separated into different classes, including non-aromatic hydrocarbons (HC), monocyclic aromatic hydrocarbons (MAHs) and polycyclic aromatic hydrocarbons (PAHs). Both substituted and non-substituted compounds could be detected, as well as analytes with homo- and heterocycles (Table S3). Furthermore, molecules containing up to four rings could be observed, such as phenanthrene and pyrene. Amongst analytes containing heteroatoms, nitrogen-containing compounds were particularly numerous. Indeed, many observed MAH and PAH homologues contained nitrogen atoms within their ring structures (hetero-MAHs and PAHs, such as pyridine, indole, quinoline, carbazole and benzoquinoline) or in external functional groups (substituted MAHs and PAHs, such as aniline and napththalenecarbonitrile).

The list of analytes was compared with those found in previous studies, and it was confirmed that most of the compounds detected in this work have also previously been reported in gunshot exhausts [11, 14]. New molecules compatible with the discharge mechanism [37, 38] could, nonetheless, be identified here for the first time, including propenylbenzene, 1,4-dihydronaphthelene, 1H-phenalene, isocyanatobenzene and thiophene. However, no aromatic compounds with five or more rings were observed, such as benzo[a]pyrene, contrary to some previous studies using different extraction techniques [11, 14]. An in-depth comparison with recent work by Gallidabino et al. [11] was specifically carried out, where the same four ammunition types were analysed using a HSSE–based method. Perhaps as expected, more analytes were observed in the earlier work (*n* = 166 vs. 120) [39], but, of the 113 analytes identified here, only 80 were previously reported by Gallidabino et al., highlighting that 33 additional compounds could solely be observed by headspace SPME and not HSSE (Table S2). This group was mainly composed of early eluting compounds (median *t*_*R*_ = 6.542 min).

These results indicated a good overlap between headspace SPME and other more exhaustive approaches in terms of the kinds of analyte that can be extracted from gunshot exhausts, whilst also proving SPME to be more suitable for the analysis of some of the most volatile and polar compounds.

### Affinity and selectivity of the four fibre coatings

The list of compounds detected was compared between fibres. It was observed that 85 of the 120 analytes (70.8%) could be recovered with any of the four fibres tested, whilst the majority of those remaining (*n* = 33, 27.5%) were extracted by at least two (Fig. S1). Just three analytes could only be detected with a single fibre. These were carbonyl sulphide by CAR, an isomer of benzoquinoline by DVB and Akardite II (1-methyl-3,3-diphenylurea) by PA. Therefore, the four fibres presented some important overlaps in the kinds of analyte that they could extract. DVB and CAR recovered the most and least compounds, respectively, compared to the other fibres (i.e. *n* = 117 and 98).

**Fig. 2:**
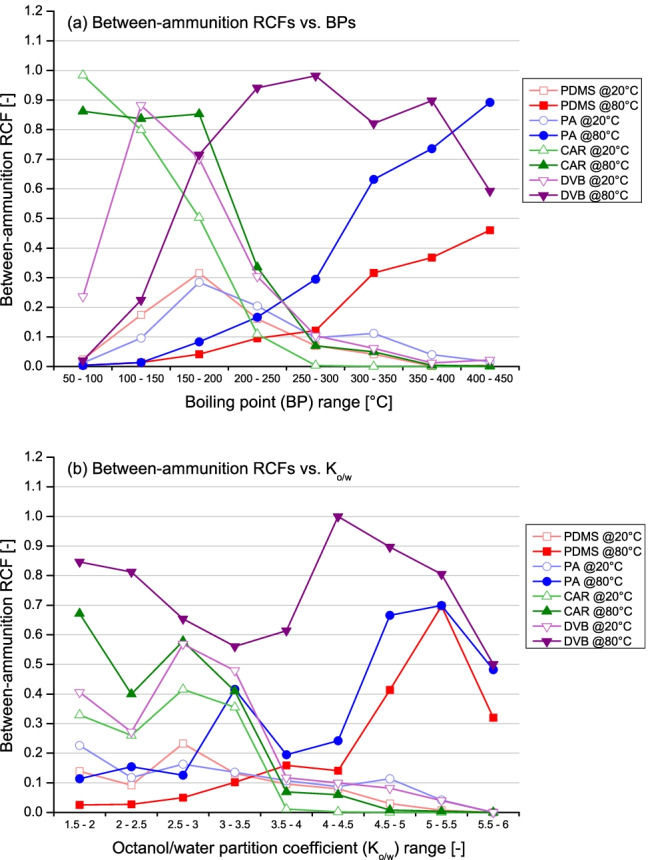
Dot plots showing the averaged between-ammunition relative concentration factors (RCFs) observed for the different extraction conditions tested in this work against (**a**) the boiling points (BP) and (**b**) octanol/water partition constants (*K*_o/w_) of the target compounds

Despite important qualitative similarities between fibres, however, a more in-depth investigation into peak areas and relative concentration factors (RCFs) revealed clear differences in the amounts recovered for almost all the observed analytes (target and non-target) (Fig. 2). In particular, CAR displayed significant uptakes across ammunition types for the compounds characterised by a high volatility and polarity (BPs = [50, 250] °C and *K*_*o/w*_ = [1.5, 3.5]), whilst PA and PDMS performed better for those characterised by a mid-low volatility and polarity (BPs = [150, 450] °C and *K*_*o/w*_ = [3.0, 6.0]). For these fibres, observed uptakes were significantly lower (or negligible) outside the ranges just described, thus also indicating their limited affinity for compounds in gunshot exhausts. This was especially true for CAR, which was found to be quite selective. DVB, on the contrary, displayed significant uptakes for analytes spanning a wide range of volatilities and polarities (BPs = [100, 450] °C and *K*_*o/w*_ = [1.5, 6.0]) and, therefore, was also observed to have a better extraction capacity compared to the other fibres tested.

Results obtained at the two extraction temperatures were compared, and as expected, differences in the extraction yields for most analytes were observed. Interestingly, however, the effect of the increase from 20 to 80 °C on the performances of the single fibres differed greatly (Fig. 2). In this regard, CAR was found to be the least affected, as it displayed good uptakes at both temperatures tested and only a marginal shift of the central maximum towards the least volatile and polar compounds. This indicated its robustness towards the extraction temperature. On the contrary, PA and PDMS generally displayed poor uptakes when a temperature of 20 °C was used and good extraction yields were only observed at 80 °C. Therefore, they were found to be much more sensitive to temperature conditions compared to CAR, emphasising the need for a careful optimisation to maximise recoveries.

Regarding DVB, its performance was also found to be significantly affected by the extraction temperature but, unlike PA and PDMS, good uptakes were observed at both conditions tested and the main difference was a shift in its apparent selectivity (Fig. 2). Specifically, DVB proved to be particularly efficient (and selective) for the recovery of compounds with a higher volatility and polarity when used at 20 °C but, at 80 °C, for those characterised by a mid-low volatility and polarity. This observation was deemed interesting, as it showed the selectivity of this fibre could be tailored by fine-tuning the extraction temperature. In particular, the extraction performances of DVB switched from being more similar to those of CAR when used at a low extraction temperature to being more similar to those of PA and PDMS when used at a high extraction temperature, albeit with a larger coverage in terms of chemical classes of compounds extracted.

### Analysis and characterisation of exhausts

For each target analyte, the results observed at the different extraction conditions were compared and this confirmed the significant effect on recoveries of both the fibre and temperature used. Analysis of variance (ANOVA), in particular, showed median *p* values ≤ 0.001 across all the target analytes for the main effects on peak areas provided by both these variables, as well as their reciprocal interaction (Table S4). Noteworthy was that the largest uptakes for each analyte were not reached using a unique set of conditions. Therefore, optimal conditions (and fibres) maximising the recovery efficiency for the different analytes were investigated (Fig. S3 and Table S5).

Perhaps as expected from previous findings, CAR was found to be the best fibre for the sensitive recovery of the majority of the most volatile and polar compounds, such as benzaldehyde and benzonitrile, whilst PA and PDMS were found to be the best fibres for the sensitive recovery of some of the least volatile and polar compounds, such as dibutyl phthalate and ethyl centralite. PA and PDMS, specifically, were found to have very similar selectivities and to be particularly efficient for the extraction of the residual smokeless-powder components. Yet, PA was generally more efficient in terms of recovery efficiencies compared to PDMS for almost all the target analytes, with ethyl centralite and 4-nitrodiphenlyamine being the only exceptions. DVB displayed the best extraction efficiencies for all analytes characterised by an intermediary volatility and polarity. Independently from the fibre used, higher uptakes were typically observed at 80 °C compared to 20 °C, but the use of a lower extraction temperature seemed to be an advantage in some situations, such as, for example, the extraction of benzene and toluene with CAR or the extraction of xylene and styrene with DVB (Fig. S3 and Table S5).

**Fig. 3:**
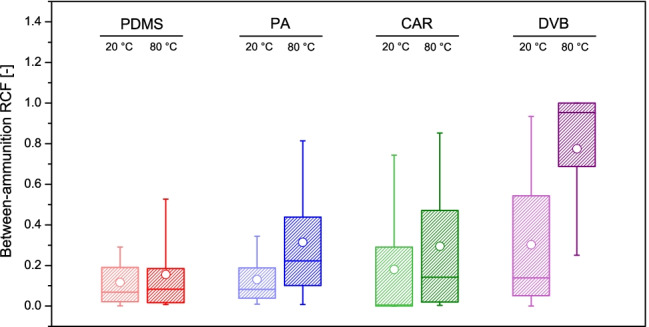
Boxplots representing the distribution of the between-ammunition relative concentration factors (RCFs) for the different target analytes detected in chromatograms (*n* = 50), as a function of the condition used during extraction

The fibres were ranked according to their recovery efficiencies, and it could be confirmed that DVB was the most exhaustive fibre for the analysis of gunshot exhausts. Indeed, use of DVB led to median between-ammunition RCFs of 0.139 and 0.953 when used at 20 °C and 80 °C, respectively, as well as to the best uptakes for the largest number of target analytes simultaneously amongst the fibres tested. At 80 °C, in particular, DVB scored as the best or second best for 33 and 9 target analytes, respectively (Fig. 3 and Table 2). This highlighted its particular suitability for implementation in characterisation studies and, more generally, in multi-residue methods. The other three fibres were more selective, displaying, therefore, good extraction yields for fewer compounds. Still, PA led to a median between-ammunition RCF of 0.315 at 80 °C and scored as the best or second best for 4 and 20 target analytes, respectively, indicating that it was the second most exhaustive fibre after DVB.

**Table 2:** Summary statistics for the assessment metrics used in this work, i.e. between-ammunition relative concentration coefficients (RCFs), between-ammunition coefficients of variations (CVs) and *J*_2_ indices

*Fibre*	*Temp*	*Between-ammunition RCF [ −]*		*Between-ammunition CV [%]*		*J*_*2*_* index [ −]*	
		*Mean (*± *SD)*	*Median (IDR)*	*Mean (*± *SD)*	*Median (IDR)*	*Mean (*± *SD)*	*Median (IDR)*
*PDMS*	*20 °C*	0.116 (± 0.119)	0.068 (0.002–0.281)	45.7 (± 31.2)	37.1 (18.0–84.7)	73 (± 57)	59 (28–129)
	*80 °C*	0.156 (± 0.213)	0.084 (0.008–0.505)	65.8 (± 34.4)	54.1 (28.8–118.2)	176 (± 187)	115 (38–381)
*PA*	*20 °C*	0.131 (± 0.123)	0.083 (0.012–0.341)	39.8 (± 28.1)	31.5 (16.1–84.0)	508 (± 559)	321 (130–1109)
	*80 °C*	0.315 (± 0.297)	0.222 (0.010–0.787)	32.7 (± 27.9)	22.4 (10.0–76.3)	365 (± 442)	204 (63–840)
*CAR*	*20 °C*	0.180 (± 0.294)	0.005 (0.000–0.735)	36.3 (± 34.5)	22.9 (10.1–79.4)	426 (± 1017)	212 (47–932)
	*80 °C*	0.295 (± 0.337)	0.142 (0.004–0.845)	35.5 (± 29.5)	21.9 (12.1–75.3)	138 (± 217)	78 (26–292)
*DVB*	*20 °C*	0.302 (± 0.332)	0.139 (0.000–0.921)	31.6 (± 20.6)	24.1 (14.2–62.5)	95 (± 134)	58 (21–188)
	*80 °C*	0.775 (± 0.309)	0.953 (0.259–1.000)	42.9 (± 27.4)	39.0 (19.3–71.6)	52 (± 49)	38 (18–96)

*SD* standard deviation,* IDR* interdecile range of the distribution of the observed values, reported using its boundaries (i.e. the 10^th^ and 90^th^ deciles)

**Fig. 4:**
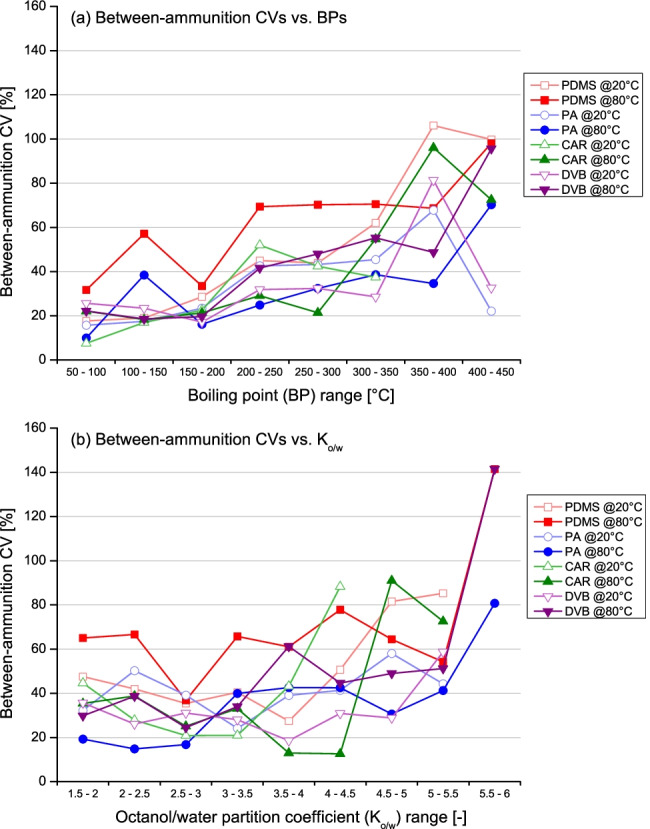
Dot plots showing the averaged between-ammunition coefficient of variations (CVs) observed for the different extraction conditions tested in this work against (**a**) the boiling point (BP) and (**b**) octanol/water partition coefficient (*K*_*o/w*_) of the target compounds

**Fig. 5:**
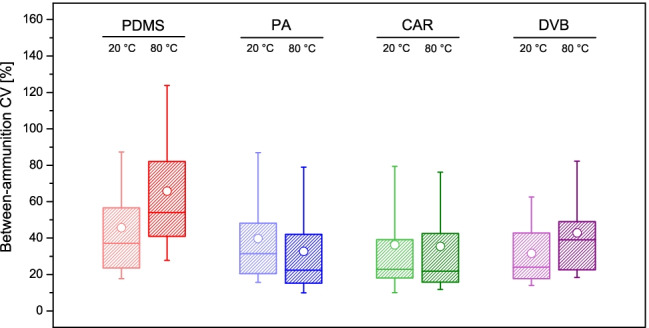
Boxplots representing the distribution of the between-ammunition coefficient of variation (CVs) for the different target analytes detected in chromatograms (*n* = 50), as a function of the condition used during extraction

The coefficients of variation (CVs) were determined within the different groups of analyses, in order to assess the expected variability of the measurements (peak areas). It could be observed that measurements were generally affected by high between-ammunition CVs (and, therefore, an overall high variability), which further increased as a function of both the BP and *K*_*o/w*_, independently from the conditions (and fibres) applied (Fig. 4). This was, however, perhaps expected and likely due to two well-known factors, which can both lead to a high variability in the amount of analyte in the headspace. The first is the random inconsistencies in the discharge conditions between different shooting events (temperature and pressure of the barrel chamber) and, the second, the particularly strong interactions between the least polar compounds and the metallic casings [10–12]. CAR typically displayed the least variable (and, thus, most reproducible) measurements amongst the four fibres tested, with a good consistency between the temperature conditions. Indeed, median between-ammunition CVs of 22.9% and 21.9% were observed at extraction temperatures of 20 °C and 80 °C, respectively (Fig. 5 and Table 2).

The other fibres showed worse performances than CAR in terms of measurement variability. Despite being the most exhaustive fibre, DVB offered poor reproducibilities between peak areas, demonstrating its limitations for (semi-)quantitative determinations. This was especially true at an extraction temperature of 80 °C (namely, the condition where it shows the largest extraction capacity), which also supported the hypothesis of important analyte competitions and displacement effects taking place on the coating during the extraction [40]. Indeed, a median between-ammunition CV of 39.0% was observed. The most variable measurements were detected with PDMS, with median between-ammunition CVs of 37.1% and 54.1% at 20 °C and 80 °C, respectively.

### Discrimination between different sources

The ability of the tested fibres to discriminate gunshot exhausts according to their source (i.e. ammunition type) is an important requirement for some applications, especially in forensic science. This has been investigated by studying the level of separation between the data clusters formed by the respective chemical profiles after principal component analysis (PCA).

**Fig. 6:**
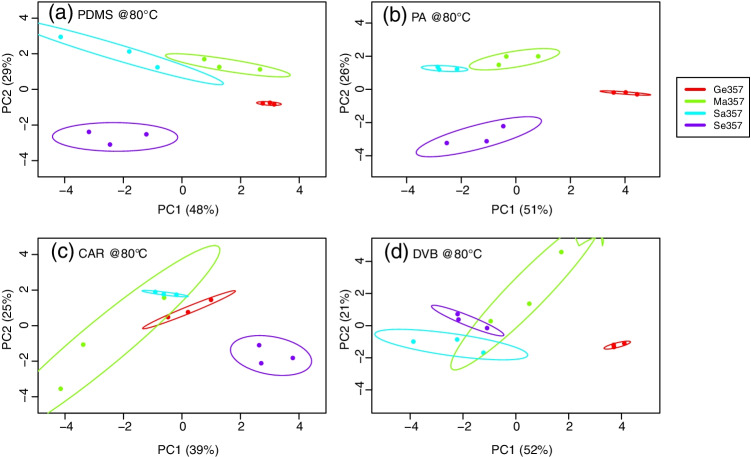
Score plots of the first two principal components (PCs) after principal component analysis (PCA) of data acquired at 80 °C using each of the four fibres tested in this work. Explained variance of each PC is reported in brackets and ellipses on graphs represent 80% confidence intervals. PCA was performed only on the selection of 14 compounds of particular interest. For the sake of comparison, *J*_2_ indices were 732 (PDMS), 2692 (PA), 95 (CAR) and 61 (DVB)

For the sake of illustration, Fig. 6 shows the score plots on the first two principal components of the chemical profiles acquired at 80 °C after PCA of a selection of 14 analytes of particular interest. It can be observed that, despite the overall high variability observed, the chemical profiles for the four ammunition types typically formed clusters in the score plots that, in most cases, allowed a discrimination between ammunition types. This was true with any of the four fibres tested, but the use of PA led to less scattered clusters and better separations compared to the others. The ammunition type was shown to have a clear effect on the quality of clustering. Ge357, indeed, provided the least scattered clusters in PCA score plots compared to the other ammunition types, whilst the inverse was true for Ma357, which typically showed the largest dispersions between its replicate analyses.

**Fig. 7:**
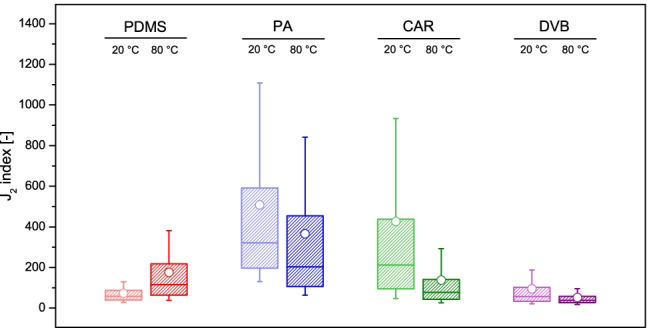
Boxplots representing the distribution of the *J*_*2*_ indices determined after principal component analysis of different subsets of target compounds (*n* = 6476), as a function of the condition used during extraction

Cluster separation in PCA score plots on the first two principal components was further investigated using the *J*_2_ criterion, which is based on the determination of a quantitative metric (i.e. the *J*_2_ index) to objectively compare the within- and between-source scatters [36]. Previous literature proved that the actual separation between clusters (i.e. ammunition types) depends on the chemical features (i.e. analytes) included in the chemical profiles. Hence, a resampling strategy was adopted, in order to test different selections of compounds. This analysis confirmed that PA generally led to the largest *J*_2_ indices and, therefore, to the best discrimination between gunshot exhausts from different sources, especially when in combination with an extraction temperature of 80 °C (Fig. 7 and Table 2). Indeed, median *J*_2_ indices of 204 and 321 were observed at 20 and 80 °C, respectively, which also supported its particular suitability for implementation in profiling approaches. CAR and PDMS did not allow the same degree of between-source discrimination as PA, but acceptable cluster separations were still observed when used at specific temperature conditions, i.e. 20 and 80 °C, respectively. At these conditions, median *J*_2_ indices of 212 and 115 were observed. In particular, the general low reproducibility observed with PDMS did not seem to largely affect its ability for between-source discrimination. DVB, on the contrary, showed the poorest discrimination performances, despite being the most exhaustive in terms of extraction recoveries.

These findings indicated that the actual discrimination power obtained with a specific fibre is not strictly related to its extraction recoveries and exhaustiveness, but it is likely the consequence of a complex interaction between them and a number of other variables (including reproducibility). Therefore, it is a characteristic that needs, preferentially, to be assessed by empirical evaluation.

## Conclusion

In this work, four different commercial SPME fibres were compared for their abilities to characterise and profile gunshot exhausts in spent cartridge casings from four different ammunition types via their headspace, as well as discriminate them according to their chemical profiles. Coatings included 100 μm polydimethylsiloxane (PDMS), 85 μm polyacrylate (PA), 65 μm polydimethylsiloxane/divinylbenzene (DVB) and 85 μm carboxen/polydimethylsiloxane (CAR).

Results showed that 120 analytes could be extracted across the different tested cartridges, but different fibres also presented distinct performances with respect to the number, type and amount of extracted compounds. In this regard, DVB proved to be the most exhaustive amongst the tested fibres, especially when used at high extraction temperatures. This was deemed particularly helpful for integration in multi-residue methods. The variability between measurements observed with this fibre, however, was generally high, making it more appropriate for characterisation purposes and/or the qualitative determination of specific compounds of interest that could not be detected with the other fibres. PA was the most suitable for broad-scope applications, due to the fact that it allowed (at least on the cartridges tested in this work) the simultaneous recovery of a large number of compounds with large uptakes, good reproducibilities and a high degree of discrimination between gunshot exhausts released from different sources to be obtained. Together, these characteristics make this fibre a good candidate for different kinds of profiling applications requiring qualitative or (semi-)quantitative assessments, such as time-since-discharge estimation and/or residue association. CAR was found to be particularly efficient for the analysis of compounds with low boiling points and high polarities, thus making it a preferential alternative to PA for the targeted analysis of this specific group of compounds. Finally, PDMS did not show any particular advantage compared to the other fibres tested.

As a conclusion, headspace SPME proved to be a valid alternative to other high-capacity and/or more exhaustive sampling approaches for the characterisation and profiling of gunshot exhausts, whilst also being simpler, less invasive and less time consuming. This is true, however, provided that the most appropriate fibre is selected depending on the specific application. This work offers useful data in this respect, making it a valuable starting point to drive the development of next-generation methods in the health, environmental and forensic science fields.

## Supplementary Information

Below is the link to the electronic supplementary material.

Supplementary file1 (DOCX 2390 KB)
