# Supplementary material for: Comparison of four commercial solid-phase micro-extraction (SPME) fibres for the headspace characterisation and profiling of gunshot exhausts in spent cartridge casings
Source: Anal Bioanal Chem. 2022 May 24;414(17):4987–98. doi: 10.1007/s00216-022-04129-w (PMC9234032; doi:10.1007/s00216-022-04129-w)
Supplement: Supplementary file 1 — Supplementary file1 (DOCX 2390 KB) [file 216_2022_4129_MOESM1_ESM.docx]

Electronic Supplementary Material (ESM) to

**Comparison of four commercial solid phase micro-extraction (SPME) fibres for the headspace characterisation and profiling of gunshot exhausts in spent cartridge casings**

Matteo D. Gallidabino*, Kelsey Bylenga, Stephanie Elliott, Rachel Irlam, Céline Weyermann

*Email: [matteo.gallidabino@northumbria.ac.uk](mailto:matteo.gallidabino@northumbria.ac.uk)

Table of contents

S-2: List of standards

S-4: List of labelled compounds

S-8: Summary of the chemical classes

S-9: Number of compounds detected with each fibre

S-10: Observed peak areas (target ions) by ammunition type

S-14: Analysis of effects by ANOVA

S-16: Between-ammunition RCFs by analyte

S-18: Performance ranking for each target analyte

S-19: Between-ammunition CVs by analyte

List of standards

**Table S1** – Analysed reference standards, with related CAS registry numbers (CAS), boiling points (BPs) and octanol-water partition coefficients (*K_o/w_*). The last column “Fibre” indicates the SPME fibre used for the extraction and injection of the related standard solutions

| ***#*** | ***Name*** | ***CAS*** | ***BP [°C]*** | ***K_o/w_ [-]*** | ***Furnisher*** | ***Purity*** | ***Fibre*** |
| --- | --- | --- | --- | --- | --- | --- | --- |
| 1 | *Acenaphthene* | 83-32-9 | 278.0 | 3.90 | Sigma | 98% | PDMS |
| 2 | *Acenaphthylene* | 208-96-8 | 280.0 | 3.90 | Aldrich | 99% | PDMS |
| 3 | *Acetophenone* | 98-86-2 | 202.0 | 1.60 | Fluka | 99% | PA |
| 4 | *Anthracene* | 120-12-7 | 350.0 | 4.50 | Alfa Aesar | 99% | PDMS |
| 5 | *Benzaldehyde* | 100-52-7 | 178.0 | 1.50 | Fluka | 99% | PA |
| 6 | *Benzene* | 71-43-2 | 80.1 | 2.00 | Fluka | 99% | PDMS |
| 7 | *Benzo[a]pyrene* | 50-32-8 | N/A | 5.52 | Sigma | 96% | PDMS |
| 8 | *Benzo[b]thiophene* | 95-15-8 | 221.0 | N/A | Alfa Aesar | 98% | PDMS |
| 9 | *Benzonitrile* | 100-47-0 | 188.0 | 1.56 | Riedel-de Haën | 99% | PA |
| 10 | *Benzophenone* | 119-61-9 | 306.0 | 3.20 | Fluka | 99% | PDMS |
| 11 | *Benzothiazole* | 95-16-9 | 227.0 | 1.97 | Alfa Aesar | 97% | PDMS |
| 12 | *Benzyl nitrile* | 140-29-4 | 233.0 | 1.60 | Fluka | 99% | PA |
| 13 | *Biphenyl* | 92-52-4 | 255.0 | 4.00 | Fluka | 98% | PA |
| 14 | *Biphenylene* | 259-79-0 | N/A | 3.90 | Aldrich | 99% | PA |
| 15 | *Carbazole* | 86-74-8 | 355.0 | 3.30 | Sigma | 95% | PDMS |
| 16 | *Chrysene* | 218-01-9 | 448.0 | 5.80 | Aldrich | 98% | PDMS |
| 17 | *Dibutyl phthalate* | 84-74-2 | 340.0 | 4.60 | Aldrich | 99% | PDMS |
| 18 | *1,2-Dicyanobenzene* | 91-15-6 | 304.6 | N/A | Aldrich | 98% | PA |
| 19 | *1,3-Dicyanobenzene* | 626-17-5 | 288.0 | N/A | Aldrich | 98% | PA |
| 20 | *1,4-Dicyanobenzene* | 623-26-7 | N/A | N/A | Aldrich | 98% | PA |
| 21 | *1,4-Dimethylnaphthalene* | 571-58-4 | 263.0 | 4.22 | Fluka | 95% | PDMS |
| 22 | *2,6-Dimethylnaphthalene* | 581-42-0 | 262.0 | 4.39 | Fluka | 98% | PA |
| 23 | *2,4-Dinitrodiphenylamine* | 961-68-2 | N/A | 3.70 | Aldrich | 98% | PA |
| 24 | *2,4-Dinitrotoluene* | 121-14-2 | N/A | N/A | Aldrich | 97% | PA |
| 25 | *2,6-Dinitrotoluene* | 606-20-2 | N/A | N/A | Aldrich | 98% | PA |
| 26 | *Diphenylamine* | 122-39-4 | 302.0 | 3.40 | Fluka | 98% | PDMS |
| 27 | *Ethylbenzene* | 100-41-4 | 136.2 | 3.15 | Alfa Aesar | 99% | PDMS |
| 28 | *Ethyl centralite* | 85-98-3 | 327.0 | N/A | Aldrich | 99% | PA |
| 29 | *2-Ethylhexanal* | 123-05-7 | N/A | N/A | Aldrich | 96% | PDMS |
| 30 | *2-Ethyl-1-hexanol* | 104-76-7 | 184.6 | 2.72 | Fluka | 99% | PDMS |
| 31 | *2-Ethylnaphthalene* | 939-27-5 | 258.0 | N/A | Fluka | 99% | PDMS |
| 32 | *Fluoranthene* | 206-44-0 | 375.0 | 5.10 | Aldrich | 98% | PDMS |
| 33 | *Fluorene* | 86-73-7 | 298.0 | 4.10 | Aldrich | 98% | PDMS |
| 34 | *Indene* | 95-13-6 | 180.0 | 2.90 | Aldrich | 99% | PDMS |
| 35 | *Indole* | 120-72-9 | 254.0 | 2.20 | Fluka | 98% | PA |
| 36 | *Isoquinoline* | 119-65-3 | 242.0 | 2.09 | Aldrich | 97% | PA |
| 37 | *4-Methylbiphenyl* | 644-08-6 | 267.0 | N/A | Alfa Aesar | 98% | PA |
| 38 | *1-Methyl-3,3-diphenylurea* | 13114-72-2 | 413.0 | N/A | Aldrich | 99% | PDMS |
| 39 | *1-Methylnaphthalene* | 90-12-0 | 241.0 | 3.90 | Aldrich | 95% | PDMS |
| 40 | *2-Mehtylnaphthalene* | 91-57-6 | 241.0 | 3.90 | Aldrich | 98% | PA |
| 41 | *Naphthalene* | 92-24-0 | 218.0 | 3.30 | Aldrich | 99% | PA |
| 42 | *1-Naphthalenecarbonitrile* | 86-53-3 | 299.0 | N/A | Alfa Aesar | 95% | PDMS |
| 43 | *2-Naphthalenecarbonitrile* | 613-46-7 | 308.0 | 2.89 | Alfa Aesar | 97% | PDMS |
| 44 | *2-Nitrodiphenylamine* | 119-75-5 | 346.0 | 3.07 | Alfa Aesar | 98% | PDMS |
| 45 | *4-Nitrodiphenylamine* | 836-30-6 | 211.0 | 3.74 | Aldrich | 99% | PA |
| 46 | *Phenanthrene* | 85-01-8 | 340.0 | 4.50 | Fluka | 97% | PDMS |
| 47 | *Pyrene* | 129-00-0 | 404.0 | 5.20 | Fluka | 99% | PDMS |
| 48 | *Quinoline* | 91-22-5 | 237.0 | 2.00 | Alfa Aesar | 98% | PDMS |
| 49 | *Styrene* | 100-42-5 | 144.0 | 3.05 | Alfa Aesar | 99% | PDMS |
| 50 | *Toluene* | 108-88-3 | 110.6 | 2.80 | Merck | 99% | PDMS |
| 51 | *m-Tolunitrile* | 620-22-4 | 213.0 | 2.12 | Aldrich | 99% | PDMS |
| 52 | *o-Tolunitrile* | 529-19-1 | 205.0 | 2.21 | Aldrich | 97% | PDMS |
| 53 | *p-Tolunitrile* | 104-85-8 | 217.0 | N/A | Aldrich | 98% | PA |
| 54 | *1,2,3-Trimethylbenzene* | 526-73-8 | N/A | N/A | Aldrich | 99% | PDMS |
| 55 | *m-Xylene* | 108-38-3 | N/A | N/A | Fluka | 98% | PDMS |
| 56 | *o-Xylene* | 95-47-6 | 144.4 | 3.12 | Fluka | 98% | PDMS |
| 57 | *p-Xylene* | 106-42-3 | 138.3 | 3.28 | Fluka | 98% | PDMS |

List of labelled compounds

**Table S2** – List of compounds identified in the analysed gunshot exhausts, with the average chromatographic retention time (*t_R_*), the method used for identification (ID) and whether the compound has been observed using each of the four fibres tested (PDMS, PA, CAR and DVB). The last column indicates whether the compound was detected by Gallidabino *et al.* [11] through analysis by headspace sorptive extraction (HSSE).

| **Explosion (by-)products** | |  |  |  |  |  |  |  |  |  |
| --- | --- | --- | --- | --- | --- | --- | --- | --- | --- | --- |
| ***#*** | ***Identity*** | ***t_R_ [min]*** | ***Target ion*** | ***ID^a^*** | ***Max MI*** | ***PDMS*** | ***PA*** | ***CAR*** | ***DVB*** | ***[11]*** |
| Other low molecular weight compounds | |  |  |  |  |  |  |  |  |  |
| 1 | *Carbonyl sulfide* | 1.357 | 60 | DB. MS | 9 |  |  | X |  |  |
| 2 | *Carbon disulfide* | 1.695 | 76 | DB. MS | 90 | X | X | X | X |  |
| Non-aromatic HC substituted with HA-containing groups | | |  |  |  |  |  |  |  |  |
| 3 | *Acetaldehyde* | 1.403 | 44 | DB. MS | 72 | X | X | X | X |  |
| 4 | *Acetonitrile* | 1.552 | 41 | DB. MS | 5 |  | X | X | X |  |
| 5 | *Propenenitrile (Acrylonitrile)* | 1.644 | 53 | DB. MS | 90 | X | X | X | X |  |
| 6 | *Propanenitrile* | 1.852 | 54 | DB. MS | 78 |  |  | X | X |  |
| 7 | *Butenenitrile isomer* | 2.345 | 41 | DB. MS | 87 |  |  | X | X |  |
| 8 | *Hexanenitrile* | 5.717 | 41 | DB. MS | 83 | X | X | X | X | X |
| 9 | *Diethylbenzoquinone isomer* | 17.645 | 177 | DB. MS | 99 | X | X | X | X | X |
| Not substituted MAHs | |  |  |  |  |  |  |  |  |  |
| 10 | *Benzene* | 2.579 | 78 | DB, MS, STD | 91 | X | X | X | X | X |
| MAHs substituted with hydrocarbyl groups | |  |  |  |  |  |  |  |  |  |
| 11 | *Toluene* | 3.925 | 91 | DB, MS, STD | 95 | X | X | X | X | X |
| 12 | *Ethylbenzene* | 5.460 | 91 | DB, MS, STD | 91 | X | X | X | X | X |
| 13 | *p-Xylene* | 5.541 | 91 | DB, MS, STD | 97 | X | X | X | X | X |
| 14 | *m-Xylene* | 5.541 | 91 | STD | N/A | X | X | X | X | X |
| 15 | *o-Xylene* | 5.962 | 91 | STD | N/A | X | X | X | X | X |
| 16 | *Styrene* | 5.982 | 104 | DB, MS, STD | 97 | X | X | X | X | X |
| 17 | *Propenylbenzene isomer* | 6.903 | 117 | DB. MS | 95 | X | X | X | X |  |
| 18 | *1,2,3-Trimethylbenzene* | 8.266 | 105 | STD | N/A | X | X | X | X |  |
| 19 | *Propynylbenzene isomer* | 8.915 | 115 | DB. MS | 90 | X | X | X | X |  |
| MAHs substituted with HA-containing groups | |  |  |  |  |  |  |  |  |  |
| 20 | *Phenyl isocyanate* | 7.128 | 119 | DB. MS | 91 | X | X | X | X |  |
| 21 | *Benzaldehyde* | 7.201 | 106 | DB, MS, STD | 97 | X | X | X | X | X |
| 22 | *Aniline* | 7.475 | 93 | DB. MS | 95 | X | X | X | X |  |
| 23 | *Phenol* | 7.526 | 94 | DB. MS | 91 |  | X |  | X |  |
| 24 | *Benzonitrile* | 7.631 | 103 | DB, MS, STD | 94 | X | X | X | X | X |
| 25 | *Acetophenone* | 9.065 | 105 | DB, MS, STD | 94 | X | X | X | X | X |
| 26 | *o-Tolunitrile* | 9.116 | 117 | DB, MS, STD | 97 | X | X | X | X | X |
| 27 | *Nitrobenzene* | 9.427 | 123 | DB. MS | 90 | X | X | X | X | X |
| 28 | *m-Tolunitrile* | 9.465 | 117 | DB, MS, STD | 97 | X | X | X | X | X |
| 29 | *p-Tolunitrile* | 9.803 | 117 | DB, MS, STD | 96 | X | X | X | X | X |
| 30 | *N-Ethylaniline* | 10.214 | 106 | DB. MS | 97 | X | X | X | X | X |
| 31 | *Benzyl nitrile* | 10.499 | 117 | DB, MS, STD | 96 | X | X | X | X | X |
| 32 | *1,4-Dicyanobenzene* | 13.122 | 128 | STD | N/A | X | X | X | X | X |
| 33 | *1,3-Dicyanobenzene* | 13.122 | 128 | STD | N/A | X | X | X | X | X |
| 34 | *1,2-Dicyanobenzene* | 14.474 | 128 | STD | N/A | X | X | X | X | X |
| 35 | *Butyl benzoate* | 15.501 | 105 | DB. MS | 93 | X | X | X | X | X |
| Non-substituted hetero-MAH | |  |  |  |  |  |  |  |  |  |
| 36 | *Thiophene* | 2.527 | 84 | DB. MS | 91 | X | X | X | X |  |
| 37 | *Pyridine* | 3.495 | 79 | DB. MS | 91 | X | X | X | X |  |
| 38 | *Pyrrole* | 3.649 | 67 | DB. MS | 50 |  | X |  | X |  |
| Hetero-MAH substituted with hydrocarbyl groups | | |  |  |  |  |  |  |  |  |
| 39 | *Methylhiophene isomer* | 3.997 | 97 | DB. MS | 94 | X | X | X | X |  |
| Hetero-MAH substituted with HA-containing groups | | |  |  |  |  |  |  |  |  |
| 40 | *3-Furaldehyde* | 4.588 | 95 | DB. MS | 64 | X |  | X |  |  |
| 41 | *2-Furaldehyde (furfural)* | 4.926 | 96 | DB. MS | 86 |  | X | X | X |  |
| Non-substituted PAH (fused) | |  |  |  |  |  |  |  |  |  |
| 42 | *Indane* | 8.449 | 117 | DB. MS | 81 | X | X | X | X | X |
| 43 | *Indene* | 8.655 | 116 | DB, MS, STD | 97 | X | X | X | X | X |
| 44 | *1,4-Dihydronaphthalene* | 10.935 | 129 | DB. MS | 93 | X | X | X | X |  |
| 45 | *Naphthalene* | 11.483 | 128 | DB, MS, STD | 97 | X | X | X | X | X |
| 46 | *Acenaphthylene* | 17.352 | 152 | DB, MS, STD | 91 | X | X | X | X | X |
| 47 | *Biphenylene* | 17.711 | 152 | STD | N/A | X | X | X | X | X |
| 48 | *Acenaphthene* | 18.333 | 154 | DB, MS, STD | 64 | X | X | X | X | X |
| 49 | *1H-Phenalene* | 19.829 | 165 | DB. MS | 87 | X | X |  | X |  |
| 50 | *Fluorene* | 20.323 | 165 | DB, MS, STD | 97 | X | X | X | X | X |
| 51 | *Phenanthrene* | 24.492 | 178 | DB, MS, STD | 96 | X | X | X | X | X |
| 52 | *Anthracene* | 24.850 | 178 | DB, MS, STD | 90 | X | X | X | X | X |
| 53 | *4H-Cyclopenta[def]phenanthrene* | 27.194 | 190 | DB. MS | 97 | X | X |  | X | X |
| 54 | *Fluoranthene* | 29.746 | 202 | DB, MS, STD | 96 | X | X | X | X | X |
| 55 | *Pyrene* | 30.607 | 202 | DB, MS, STD | 96 | X | X | X | X | X |
| 56 | *Chrysene* | 34.407 | 228 | STD | N/A | X | X |  | X | X |
| PAH (fused) substituted with hydrocarbyl groups | | |  |  |  |  |  |  |  |  |
| 57 | *2-Methylnaphthalene* | 13.931 | 142 | DB, MS, STD | 97 | X | X | X | X | X |
| 58 | *1-Methylnaphthalene* | 14.136 | 142 | DB, MS, STD | 95 | X | X | X | X | X |
| 59 | *2-Ethylnaphthalene* | 16.000 | 141 | STD | N/A | X | X | X | X | X |
| 60 | *Dimethylnaphthalene isomer* | 16.170 | 156 | DB. MS | 50 | X | X | X | X | X |
| 61 | *2,6-Dimethylnaphthalene* | 16.512 | 156 | STD | N/A | X | X | X | X | X |
| 62 | *Dimethylnaphthalene isomer* | 16.573 | 156 | DB. MS | 97 | X | X | X | X | X |
| 63 | *Ethenylnaphthalene isomer* | 16.783 | 154 | DB. MS | 96 | X | X | X | X | X |
| 64 | *Dimethylnaphthalene isomer* | 17.016 | 156 | DB. MS | 97 | X | X |  | X | X |
| 65 | *1,4-Dimethylnaphthalene* | 17.045 | 156 | STD | N/A | X | X | X | X | X |
| 66 | *Diisopropylnaphthalene isomer* | 22.280 | 197 | DB. MS | 93 | X | X | X | X | X |
| 67 | *Diisopropylnaphthalene isomer* | 22.396 | 197 | DB. MS | 95 | X | X | X | X | X |
| 68 | *Phenylnaphthalene isomer* | 26.086 | 204 | DB. MS | 94 | X | X |  | X | X |
| PAH (fused) substituted with HA-containing groups | | |  |  |  |  |  |  |  |  |
| 69 | *1-Naphthalenecarbonitrile* | 18.448 | 153 | DB, MS, STD | 97 | X | X | X | X | X |
| 70 | *2-Naphthalenecarbonitrile* | 19.032 | 153 | DB, MS, STD | 95 | X | X | X | X | X |
| Non-substituted PAH (joined) | |  |  |  |  |  |  |  |  |  |
| 71 | *Biphenyl* | 15.898 | 154 | DB, MS, STD | 94 | X | X | X | X | X |
| 72 | *Diphenylmethane* | 16.873 | 168 | DB. MS | 94 | X |  |  | X | X |
| PAH (joined) substituted with hydrocarbyl groups | | |  |  |  |  |  |  |  |  |
| 73 | *4-Methylbiphenyl* | 18.612 | 168 | STD | N/A | X | X | X | X | X |
| 74 | *Ethenylbiphenyl isomer* | 20.836 | 180 | DB. MS | 90 | X | X |  | X |  |
| PAH,(joined) substituted with HA-containing groups | | |  |  |  |  |  |  |  |  |
| 76 | *Diphenyl sulfide* | 20.337 | 186 | DB. MS | 83 | X | X |  | X |  |
| 75 | *Benzophenone* | 21.378 | 105 | DB, MS, STD | 95 | X | X | X | X | X |
| Non-substituted hetero-PAH (fused) | |  |  |  |  |  |  |  |  |  |
| 77 | *Benzo[b]thiophene* | 11.575 | 134 | DB, MS, STD | 95 | X | X | X | X | X |
| 78 | *Benzothiazole* | 12.282 | 135 | STD | N/A | X | X | X | X | X |
| 79 | *Quinoline* | 12.466 | 129 | DB, MS, STD | 97 | X | X | X | X | X |
| 80 | *Isoquinoline* | 13.019 | 129 | STD | N/A | X | X | X | X | X |
| 81 | *Indole* | 13.859 | 117 | STD | N/A | X | X | X | X | X |
| 82 | *Dibenzofuran* | 18.752 | 168 | DB. MS | 81 | X | X |  | X | X |
| 83 | *Benzoquinoline isomer* | 21.742 | 179 | DB. MS | 95 | X | X | X | X | X |
| 84 | *Acridine* | 22.937 | 179 | DB. MS | 95 | X | X | X | X |  |
| 85 | *Benzoquinoline isomer* | 23.320 | 179 | DB. MS | 81 |  |  |  | X | X |
| 86 | *Phenazine* | 23.532 | 180 | DB. MS | 96 | X | X |  | X | X |
| 87 | *Dibenzothiophene* | 23.889 | 184 | DB. MS | 97 | X | X | X | X | X |
| 88 | *Carbazole* | 25.923 | 167 | DB, MS, STD | 91 | X | X | X | X | X |
| Hetero-PAH (fused) substituted with hydrocarbyl groups | | |  |  |  |  |  |  |  |  |
| 89 | *Methylquinoline isomer* | 14.039 | 143 | DB. MS | 97 | X | X |  | X | X |
| 90 | *Ethylcarbazole isomer* | 25.981 | 180 | DB. MS | 95 | X | X |  | X | X |
| **Smokeless powder additives** | |  |  |  |  |  |  |  |  |  |
| ***#*** | ***Identity*** | ***t_R_ [min]*** | ***Target ion*** | ***ID*** | ***Max MI*** | ***PDMS*** | ***PA*** | ***CAR*** | ***DVB*** | ***[11]*** |
| Non-aromatic HC substituted with HA-containing groups | | |  |  |  |  |  |  |  |  |
| 91 | *2-Ethyl-1-hexanol* | 8.328 | 57 | DB, MS, STD | 90 | X | X | X | X | X |
| MAH substituted with HA-containing groups | |  |  |  |  |  |  |  |  |  |
| 92 | *Diethyl phthalate* | 20.568 | 149 | DB. MS | 95 | X | X |  | X | X |
| 93 | *Dibutyl phthalate* | 28.302 | 149 | DB, MS, STD | 94 | X | X | X | X | X |
| PAH (joined) substituted with HA-containing groups | | |  |  |  |  |  |  |  |  |
| 94 | *Diphenylamine* | 21.224 | 169 | DB, MS, STD | 81 | X | X | X | X | X |
| 95 | *Ethyl centralite* | 26.981 | 120 | DB, MS, STD | 99 | X | X | X | X | X |
| 96 | *2-Nitrodiphenylamine* | 28.302 | 214 | DB, MS, STD | 99 | X | X |  | X | X |
| 97 | *1-Methyl-3,3-diphenylurea* | 29.357 | 169 | STD | N/A |  | X |  |  | X |
| 98 | *4-Nitrodiphenylamine* | 32.164 | 214 | STD | N/A | X | X |  | X | X |
| **Ambiguous origin** | |  |  |  |  |  |  |  |  |  |
| ***#*** | ***Identity*** | ***t_R_ [min]*** | ***Target ion*** | ***ID*** | ***Max MI*** | ***PDMS*** | ***PA*** | ***CAR*** | ***DVB*** | ***[11]*** |
| Non-aromatic non-substituted HC | |  |  |  |  |  |  |  |  |  |
| 99 | *Hexene isomer* | 1.901 | 56 | DB. MS | 91 | X |  | X | X |  |
| 100 | *Nonane* | 6.045 | 57 | DB. MS | 94 |  | X |  | X |  |
| 101 | *Tetradecane* | 16.015 | 57 | DB. MS | 96 | X |  | X | X | X |
| 102 | *Heptadecane* | 22.721 | 57 | DB. MS | 96 | X |  |  | X | X |
| Non-aromatic HC substituted with hydrocarbyl groups | | |  |  |  |  |  |  |  |  |
| 103 | *Methyleneheptane isomer* | 4.127 | 70 | DB. MS | 94 | X |  | X | X |  |
| Non-aromatic HC substituted with HA-containing groups | | |  |  |  |  |  |  |  |  |
| 104 | *Ethanol* | 1.481 | 45 | DB. MS | 90 | X | X |  | X |  |
| 105 | *Methyl isobutyl ketone* | 3.392 | 43 | DB. MS | 87 |  | X | X | X |  |
| 106 | *Hexanal* | 4.330 | 56 | DB. MS | 94 | X | X | X | X | X |
| 107 | *Butyl acetate* | 4.592 | 43 | DB. MS | 86 | X | X | X | X |  |
| 108 | *Heptanone isomer* | 5.839 | 57 | DB. MS | 86 | X | X | X | X | X |
| 109 | *Heptanal* | 6.101 | 44 | DB. MS | 90 |  |  | X | X | X |
| 110 | *Butoxyethanol isomer* | 6.181 | 57 | DB. MS | 87 |  | X | X | X |  |
| 111 | *2-Ethylhexanal* | 7.047 | 72 | STD | N/A | X | X | X | X |  |
| 112 | *Nonanal* | 9.662 | 57 | DB. MS | 91 |  |  | X | X | X |
| 113 | *Ethylhexyl acetate isomer* | 10.568 | 43 | DB. MS | 90 | X | X | X | X |  |
| Not labelled | |  |  |  |  |  |  |  |  |  |
| 114 | *Unknown* | 6.139 | 133 | None | N/A | X | X | X | X | N/A |
| 115 | *Unknown* | 7.066 | 193 | None | N/A | X | X | X | X | N/A |
| 116 | *Unknown* | 9.819 | 267 | None | N/A | X | X | X | X | N/A |
| 117 | *Unknown* | 14.811 | 138 | None | N/A | X | X | X | X | N/A |
| 118 | *Unknown* | 16.397 | 153 | None | N/A | X | X | X | X | N/A |
| 119 | *Unknown* | 16.652 | 66 | None | N/A | X | X | X | X | N/A |
| 120 | *Unknown* | 18.068 | 168 | None | N/A | X | X | X | X | N/A |

^a^ Method used for identification; “DB” = library comparison in NIST08 database (in this case, “Max MI” indicates the maximum match index value obtained amongst cartridges, on a scale from 0 to 100), “MS”= mass spectra analysis, “STD” = direct comparison with a reference standard.

Summary of the chemical classes

**Table S3 –** Number of compounds detected in gunshot exhausts across ammunition types, sorted by chemical classes and supposed origin, and related percentages over the total number.

| ***Chemical class^a^*** | | ***Number*** | | | | ***Percentage [%]*** | | | |
| --- | --- | --- | --- | --- | --- | --- | --- | --- | --- |
|  |  | ***Overall*** | ***Explosion products*** | ***SLP compounds*** | ***Ambiguous*** | ***Overall*** | ***Explosion products*** | ***SLP compounds*** | ***Ambiguous.*** |
| *Small molecules* | | 2 | 2 | 0 | 0 | 1.7 | 1.7 | 0.0 | 0.0 |
| *Aliphatics* | | 23 | 7 | 1 | 15 | 19.2 | 5.8 | 0.8 | 12.5 |
|  | *HC (non-substituted)* | 4 | 0 | 0 | 4 | 3.3 | 0.0 | 0.0 | 3.3 |
|  | *HC (substituted w/ hydrocarbyls)* | 1 | 0 | 0 | 1 | 0.8 | 0.0 | 0.0 | 0.8 |
|  | *HC (substituted w/ HA-based groups)* | 18 | 7 | 1 | 10 | 15.0 | 5.8 | 0.8 | 8.3 |
| *Aromatics* | | 88 | 81 | 7 | 0 | 73.3 | 67.5 | 5.8 | 0.0 |
|  | *MAH (non-substituted)* | 1 | 1 | 0 | 0 | 0.8 | 0.8 | 0.0 | 0.0 |
|  | *MAH (substituted w/ hydrocarbyls)* | 9 | 9 | 0 | 0 | 7.5 | 7.5 | 0.0 | 0.0 |
|  | *MAH (substituted w/ HA-based groups)* | 18 | 16 | 2 | 0 | 15.0 | 13.3 | 1.7 | 0.0 |
|  | *Hetero-MAH (non-substituted)* | 3 | 3 | 0 | 0 | 2.5 | 2.5 | 0.0 | 0.0 |
|  | *Hetero-MAH (substituted w/ hydrocarbyls)* | 1 | 1 | 0 | 0 | 0.8 | 0.8 | 0.0 | 0.0 |
|  | *Hetero-MAH (substituted w/ HA-based groups)* | 2 | 2 | 0 | 0 | 1.7 | 1.7 | 0.0 | 0.0 |
|  | *PAH, fused (non-substituted)* | 15 | 15 | 0 | 0 | 12.5 | 12.5 | 0.0 | 0.0 |
|  | *PAH, fused (substituted w/ hydrocarbyls)* | 12 | 12 | 0 | 0 | 10.0 | 10.0 | 0.0 | 0.0 |
|  | *PAH, fused (substituted w/ HA-based groups)* | 2 | 2 | 0 | 0 | 1.7 | 1.7 | 0.0 | 0.0 |
|  | *PAH, joined (non-substituted)* | 2 | 2 | 0 | 0 | 1.7 | 1.7 | 0.0 | 0.0 |
|  | *PAH, joined (substituted w/ hydrocarbyls)* | 2 | 2 | 0 | 0 | 1.7 | 1.7 | 0.0 | 0.0 |
|  | *PAH, joined (substituted w/ HA-based groups)* | 7 | 2 | 5 | 0 | 5.8 | 1.7 | 4.2 | 0.0 |
|  | *Hetero-PAH (non-substituted)* | 12 | 12 | 0 | 0 | 10.0 | 10.0 | 0.0 | 0.0 |
|  | *Hetero-PAH (substituted with hydrocarbyls)* | 2 | 2 | 0 | 0 | 1.7 | 1.7 | 0.0 | 0.0 |
| *Unknown* | | 7 | 0 | 0 | 7 | 5.8 | 0.0 | 0.0 | 5.8 |
| ***TOTAL*** | | **120** | **90** | **8** | **22** | **100.0** | **75.0** | **6.7** | **18.3** |

^a^ HC = non-aromatic hydrocarbon, MAH = monocyclic aromatic hydrocarbon, PAH = polycyclic aromatic hydrocarbon

Number of compounds detected with each fibre


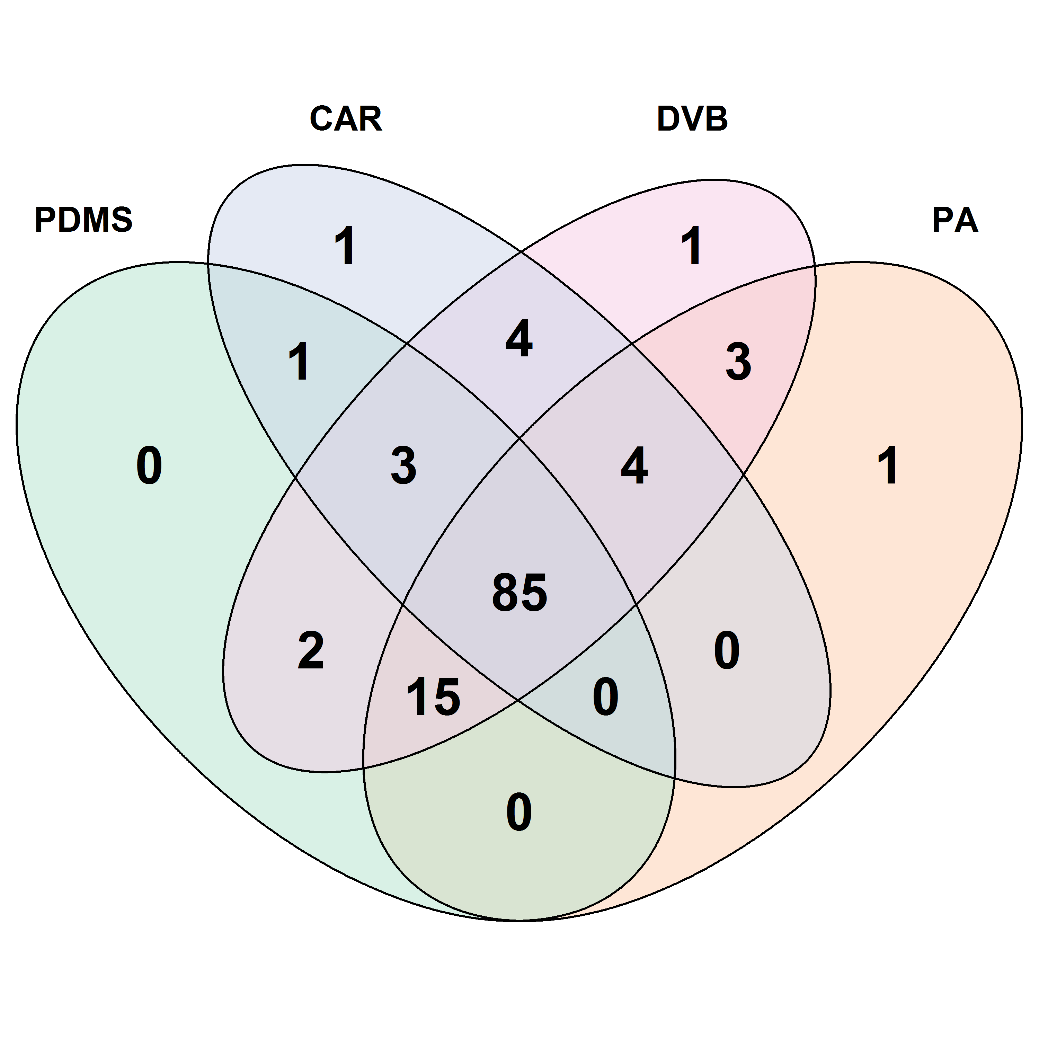


**Figure S1** – Venn diagrams reporting the number of different compounds detected in TICs between the different ammunition types by the four SPME fibres tested in this work.

Observed peak areas (target ions) by ammunition type

Ge357

**
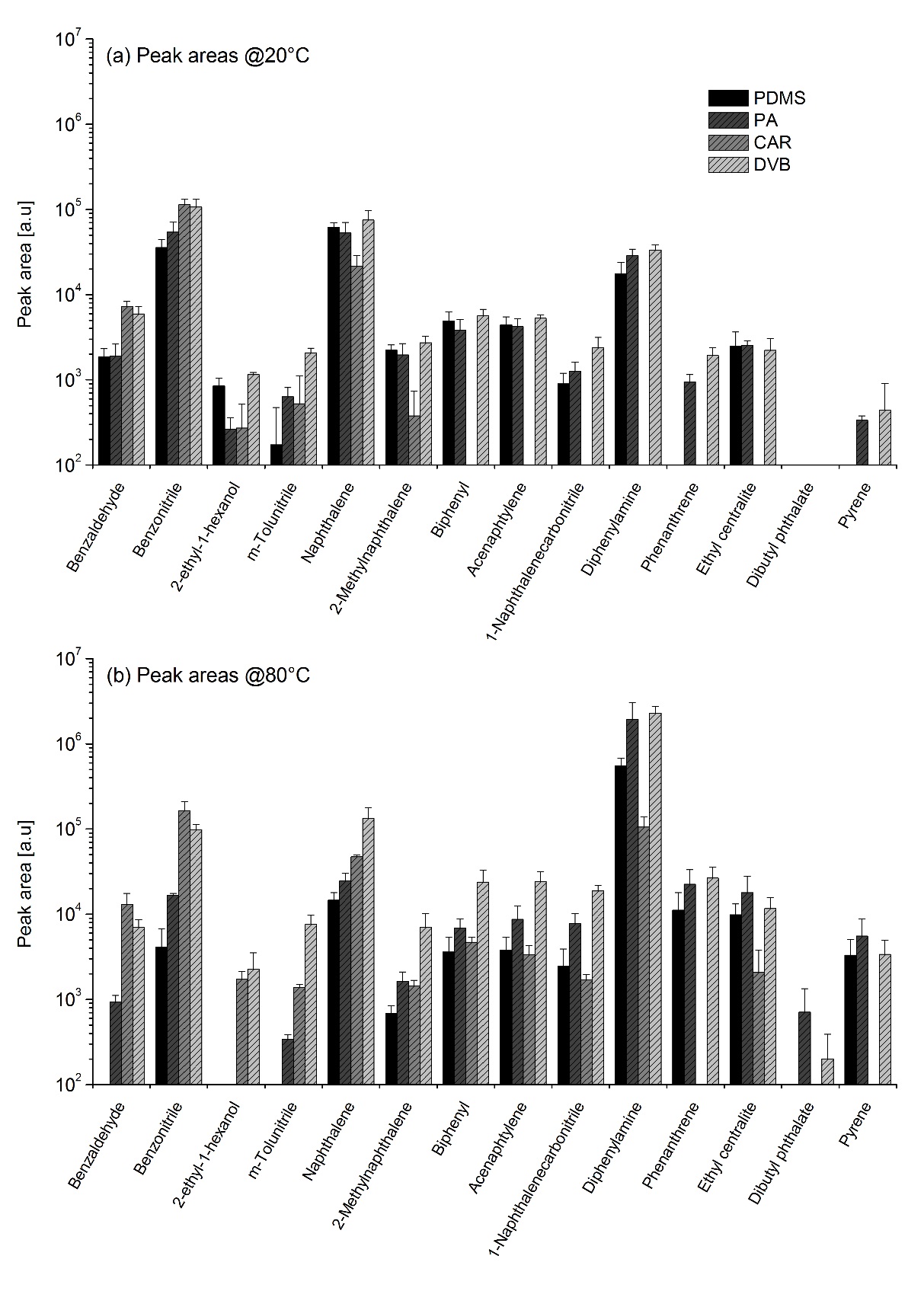
**

**Figure S2** – Bar plots comparing the peak areas (target ions, *n* = 3) observed at the different extraction conditions tested in this work for 14 compounds of particular interest (10 explosion by-products and four residual ammunition components) on the cartridges analysed at those conditions. Graphs are separated by ammunition type. Values given in base-10 log scale, in order to improve readability.

Ma357

**
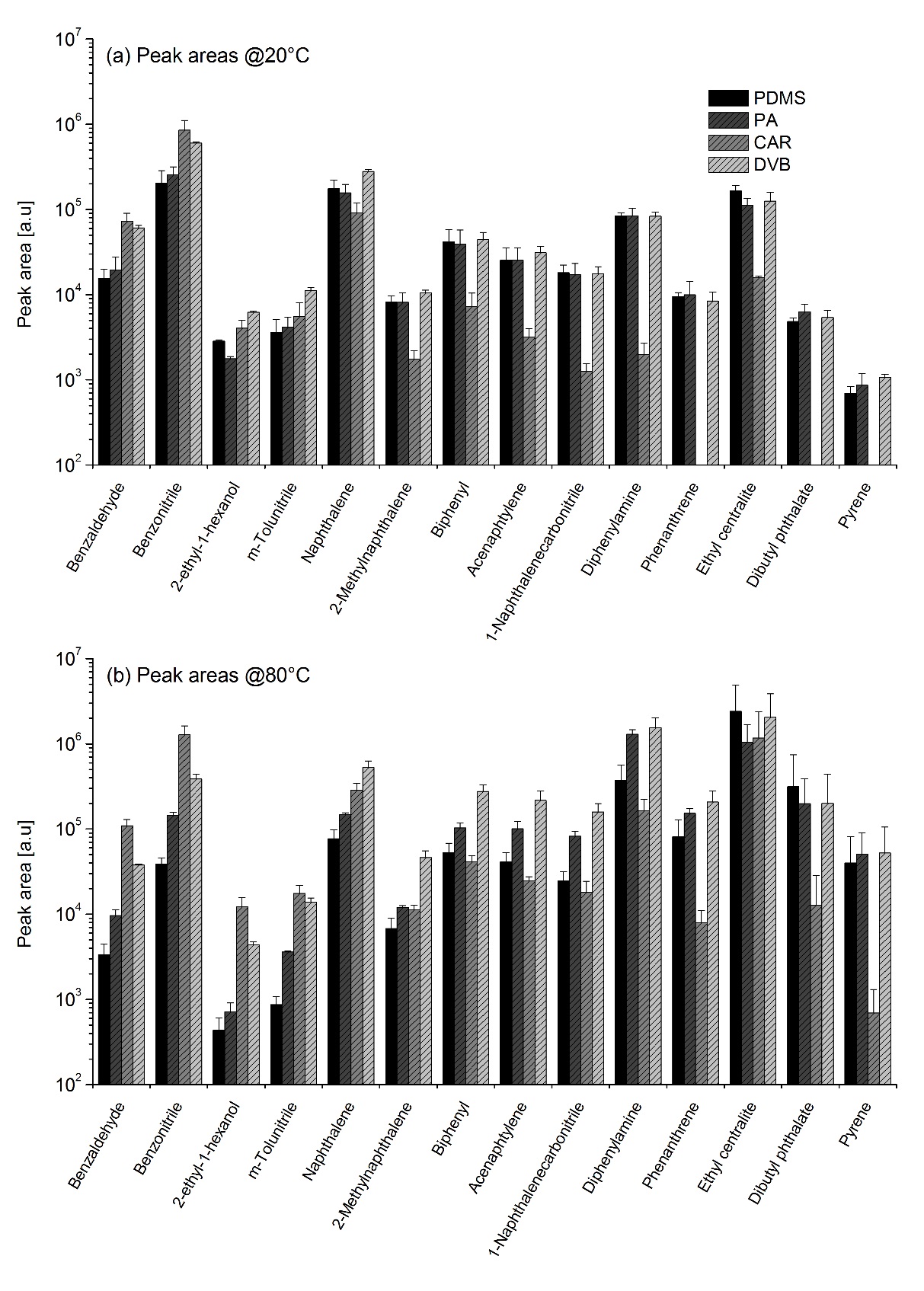
**

**Figure S2 (cont.)** – Bar plots comparing the peak areas (target ions, *n* = 3) observed at the different extraction conditions tested in this work for 14 compounds of particular interest (10 explosion by-products and four residual ammunition components) on the cartridges analysed at those conditions. Graphs are separated by ammunition type. Values given in base-10 log scale, in order to improve readability.

Sa357

**
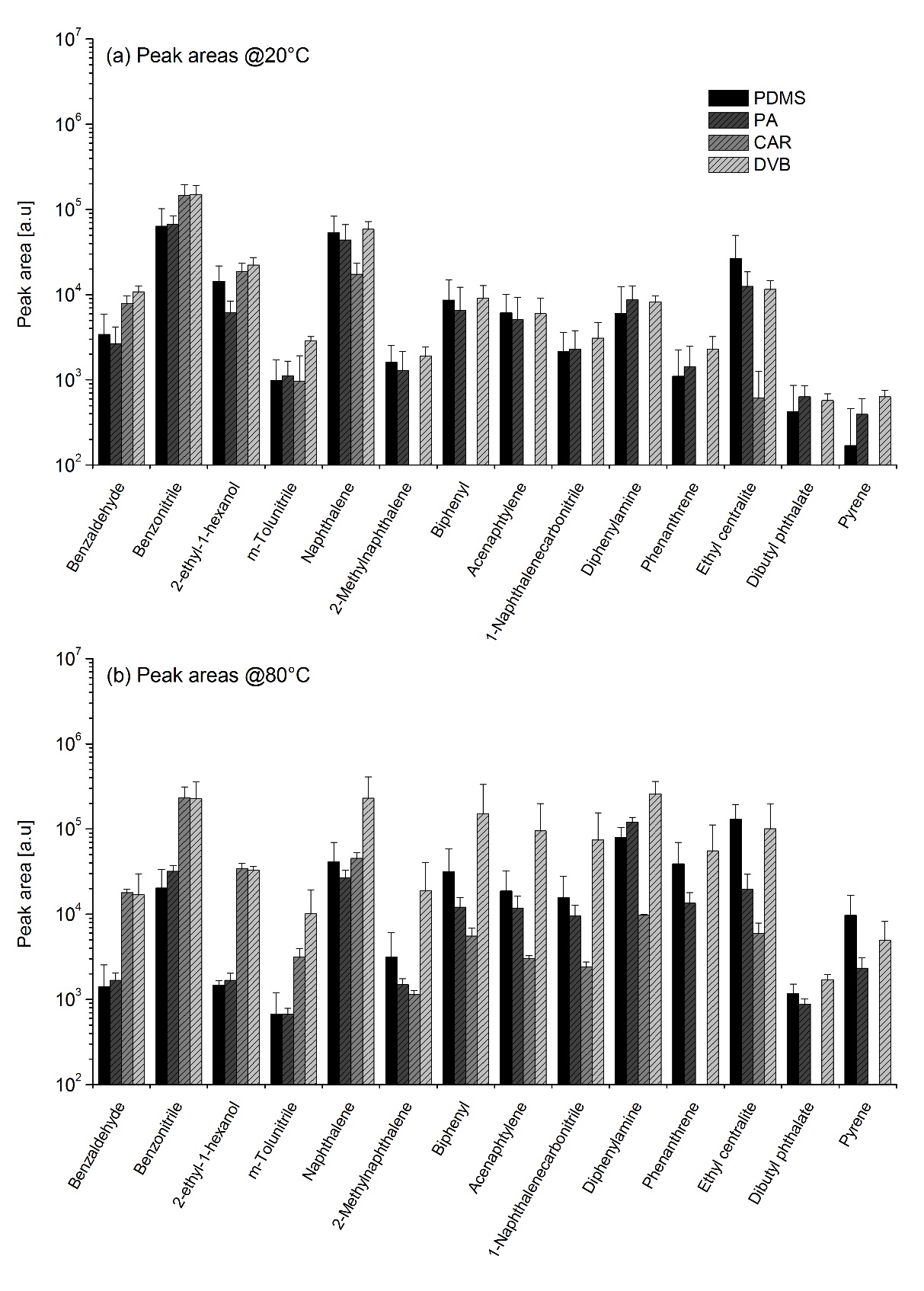
**

**Figure S2 (cont.)** – Bar plots comparing the peak areas (target ions, *n* = 3) observed at the different extraction conditions tested in this work for 14 compounds of particular interest (10 explosion by-products and four residual ammunition components) on the cartridges analysed at those conditions. Graphs are separated by ammunition type. Values given in base-10 log scale, in order to improve readability.

Se357

**
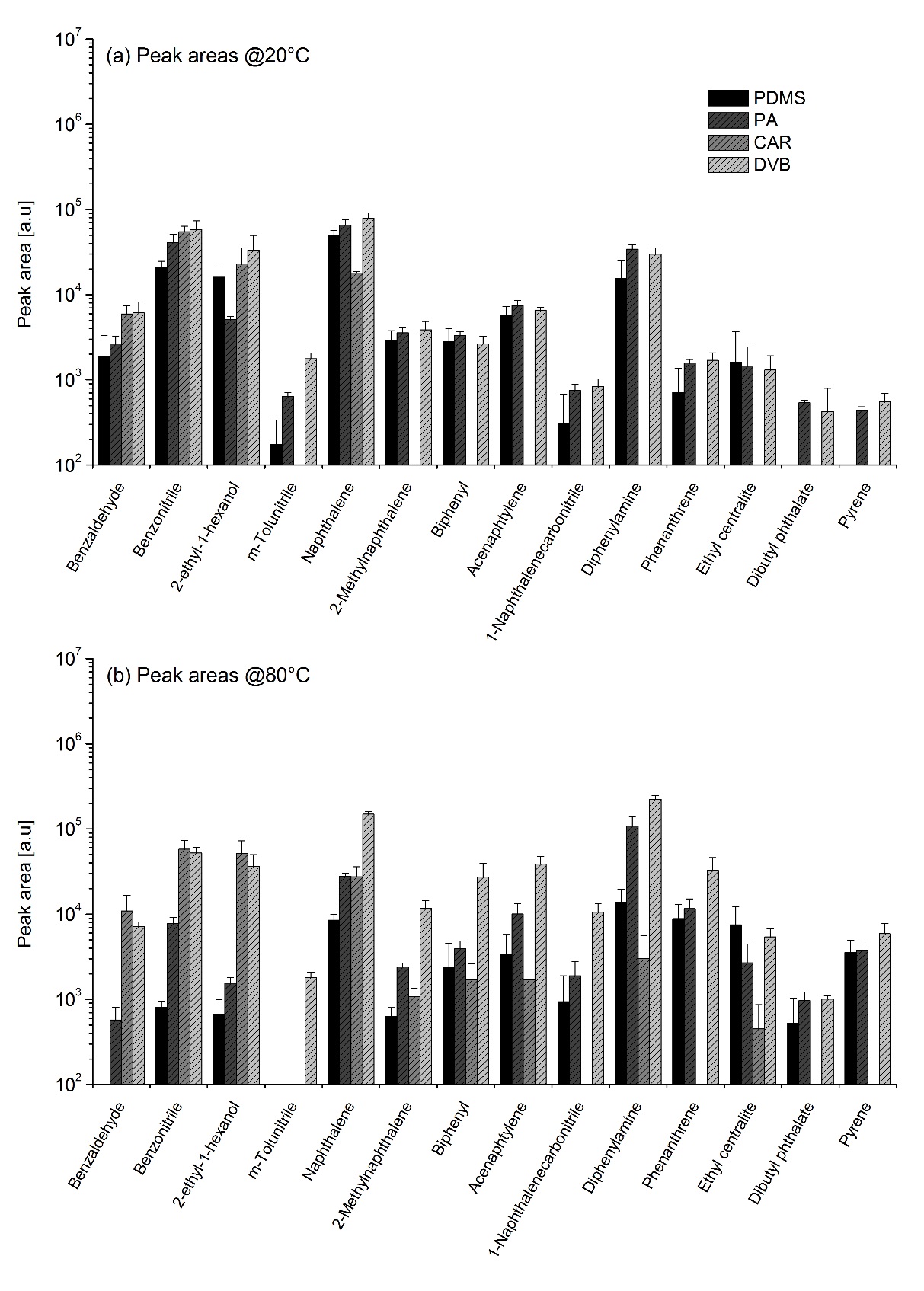
**

**Figure S2 (cont.)** – Bar plots comparing the peak areas (target ions, *n* = 3) observed at the different extraction conditions tested in this work for 14 compounds of particular interest (10 explosion by-products and four residual ammunition components) on the cartridges analysed at those conditions. Graphs are separated by ammunition type. Values given in base-10 log scale, in order to improve readability.

Analysis of effects by ANOVA

**Table S4** – p-Values for ANOVA F-tests assessing the effect of the fibre type (F), extraction temperature (T), ammunition type (A) and fibre/temperature interaction (FxT) on the uptake of the selected target compounds in headspace SPME. p-Values close to 0 support the hypothesis that any variation in the considered factor significantly affect the observed peak areas (target ions), whilst p-values close to 1 support the hypothesis that the factor does not significantly affect observed peak area. p-Values < 0.05 are reported in bold and shaded in grey.

|  | ***Compound*** | ***p-values*** | | | |
| --- | --- | --- | --- | --- | --- |
|  |  | ***Fibre (F)*** | ***Temp. (T)*** | ***Ammo (A)*** | ***FxT interaction*** |
| *1* | *Benzene* | **< 0.001** | **0.020** | **< 0.001** | 0.176 |
| *2* | *Toluene* | **< 0.001** | **< 0.001** | **< 0.001** | **< 0.001** |
| *3* | *Ethylbenzene* | **< 0.001** | **< 0.001** | **< 0.001** | **< 0.001** |
| *4* | *p-Xylene* | **< 0.001** | **0.001** | **< 0.001** | **< 0.001** |
| *5* | *o-Xylene* | **< 0.001** | **< 0.001** | **< 0.001** | **< 0.001** |
| *6* | *Styrene* | **< 0.001** | **0.016** | **< 0.001** | **0.006** |
| *7* | *2-Ethylhexanal* | **< 0.001** | **< 0.001** | **< 0.001** | **0.048** |
| *8* | *Benzaldehyde* | **< 0.001** | 0.821 | **< 0.001** | 0.081 |
| *9* | *Benzonitrile* | **< 0.001** | 0.911 | **< 0.001** | 0.151 |
| *10* | *1,2,3-Trimethylbenzene* | **< 0.001** | 0.050 | **< 0.001** | **0.004** |
| *11* | *2-Ethyl-1-hexanol* | **< 0.001** | 0.366 | **< 0.001** | **< 0.001** |
| *12* | *Indene* | **< 0.001** | **< 0.001** | **< 0.001** | **< 0.001** |
| *13* | *Acetophenone* | **< 0.001** | **0.010** | **< 0.001** | **0.010** |
| *14* | *o-Tolunitrile* | **< 0.001** | **0.192** | **< 0.001** | **0.003** |
| *15* | *m-Tolunitrile* | **< 0.001** | **0.007** | **< 0.001** | **0.002** |
| *16* | *p-Tolunitrile* | **< 0.001** | **0.009** | **< 0.001** | **0.004** |
| *17* | *Benzyl nitrile* | **< 0.001** | **0.003** | **< 0.001** | **0.011** |
| *18* | *Naphthalene* | **< 0.001** | **0.008** | **< 0.001** | **< 0.001** |
| *19* | *Benzo[b]thiophene* | **< 0.001** | **0.042** | **< 0.001** | **< 0.001** |
| *20* | *Benzothiazole* | **< 0.001** | **< 0.001** | **< 0.001** | **< 0.001** |
| *21* | *Quinoline* | **< 0.001** | **< 0.001** | **< 0.001** | **< 0.001** |
| *22* | *Isoquinoline* | **0.006** | **< 0.001** | **< 0.001** | **< 0.001** |
| *23* | *1,3-Dicyanobenzene* | **0.003** | **< 0.001** | **< 0.001** | **0.010** |
| *24* | *Indole* | **0.005** | **< 0.001** | **< 0.001** | **0.037** |
| *25* | *2-Methylnaphthalene* | **< 0.001** | **< 0.001** | **< 0.001** | **< 0.001** |
| *26* | *1-Methylnaphthalene* | **< 0.001** | **< 0.001** | **< 0.001** | **< 0.001** |
| *27* | *1,2-Dicyanobenzene* | **0.008** | **< 0.001** | **< 0.001** | **0.017** |
| *28* | *Biphenyl* | **< 0.001** | **< 0.001** | **< 0.001** | **< 0.001** |
| *29* | *2-Ethylnaphthalene* | **< 0.001** | **< 0.001** | **< 0.001** | **< 0.001** |
| *30* | *2,6-Dimethylnaphthalene* | **< 0.001** | **< 0.001** | **< 0.001** | **< 0.001** |
| *31* | *1,4-Dimethylnaphthalene* | **< 0.001** | **< 0.001** | **< 0.001** | **< 0.001** |
| *32* | *Acenaphtylene* | **< 0.001** | **< 0.001** | **< 0.001** | **< 0.001** |
| *33* | *Acenaphthene* | **< 0.001** | **< 0.001** | **< 0.001** | **< 0.001** |
| *34* | *1-Naphthalenecarbonitrile* | **< 0.001** | **< 0.001** | **< 0.001** | **< 0.001** |
| *35* | *4-Methylbiphenyl* | **< 0.001** | **< 0.001** | **< 0.001** | **< 0.001** |
| *36* | *2-Naphthalenecarbonitrile* | **< 0.001** | **< 0.001** | **< 0.001** | **< 0.001** |
| *37* | *Fluorene* | **< 0.001** | **< 0.001** | **< 0.001** | **< 0.001** |
| *38* | *Diphenylamine* | **< 0.001** | **< 0.001** | **< 0.001** | **< 0.001** |
| *39* | *Benzophenone* | **< 0.001** | **< 0.001** | **< 0.001** | **< 0.001** |
| *40* | *Phenanthrene* | **< 0.001** | **< 0.001** | **< 0.001** | **0.002** |
| *41* | *Anthracene* | **0.004** | **< 0.001** | **< 0.001** | **0.011** |
| *42* | *Carbazole* | 0.094 | **0.001** | **< 0.001** | 0.108 |
| *43* | *Ethyl centralite* | 0.656 | **0.002** | **< 0.001** | 0.758 |
| *44* | *2-Nitrodiphenylamine* | 0.566 | **0.014** | **0.001** | 0.566 |
| *45* | *Dibutyl phthalate* | 0.581 | **0.025** | **0.001** | 0.614 |
| *46* | *Methyldiphenylurea* | **0.003** | **0.029** | **0.036** | **0.003** |
| *47* | *Fluoranthene* | 0.272 | **0.001** | **< 0.001** | 0.302 |
| *48* | *Pyrene* | 0.186 | **< 0.001** | **< 0.001** | 0.239 |
| *49* | *4-Nitrodiphenylamine* | 0.689 | 0.109 | 0.277 | 0.689 |
| *50* | *Chrysene* | 0.681 | **0.047** | **0.008** | 0.681 |
|  |  |  |  |  |  |
|  | *MEAN* | 0.075 | 0.054 | **0.006** | 0.091 |
|  | *MEDIAN* | **< 0.001** | **< 0.001** | **< 0.001** | **0.001** |

Between-ammunition RCFs by analyte

**Table S5** – Between-ammunition relative concentration factors (RCFs) observed at each extraction condition. These were obtained by averaging the RCFs determined after the extraction of each ammunition type (*n* = 4).

| ***Compound*** | | ***Between-ammunition RCF [-]*** | | | | | | | |
| --- | --- | --- | --- | --- | --- | --- | --- | --- | --- |
|  |  | ***PDMS*** | | ***PA*** | | ***CAR*** | | ***DVB*** | |
|  |  | ***@20°C*** | ***@80°C*** | ***@20°C*** | ***@80°C*** | ***@20°C*** | ***@80°C*** | ***@20°C*** | ***@80°C*** |
| *1* | *Benzene* | 0.024 | 0.004 | 0.012 | 0.003 | 0.984 | 0.862 | 0.236 | 0.020 |
| *2* | *Toluene* | 0.067 | 0.009 | 0.035 | 0.007 | 0.936 | 0.884 | 0.648 | 0.066 |
| *3* | *Ethylbenzene* | 0.202 | 0.017 | 0.085 | 0.014 | 0.732 | 0.834 | 0.997 | 0.232 |
| *4* | *p-Xylene* | 0.145 | 0.010 | 0.081 | 0.010 | 0.754 | 0.822 | 0.875 | 0.239 |
| *5* | *o-Xylene* | 0.227 | 0.011 | 0.100 | 0.007 | 0.763 | 0.803 | 0.917 | 0.262 |
| *6* | *Styrene* | 0.227 | 0.020 | 0.179 | 0.027 | 0.810 | 0.843 | 0.974 | 0.320 |
| *7* | *2-Ethylhexanal* | 0.279 | 0.000 | 0.063 | 0.000 | 0.451 | 0.444 | 1.000 | 0.291 |
| *8* | *Benzaldehyde* | 0.162 | 0.027 | 0.179 | 0.076 | 0.549 | 1.000 | 0.544 | 0.621 |
| *9* | *Benzonitrile* | 0.252 | 0.039 | 0.380 | 0.121 | 0.733 | 1.000 | 0.688 | 0.692 |
| *10* | *1,2,3-Trimethylbenzene* | 0.191 | 0.000 | 0.059 | 0.000 | 0.123 | 0.283 | 0.952 | 0.389 |
| *11* | *2-Ethyl-1-hexanol* | 0.334 | 0.023 | 0.135 | 0.034 | 0.360 | 0.942 | 0.576 | 0.757 |
| *12* | *Indene* | 0.510 | 0.076 | 0.443 | 0.101 | 0.370 | 0.471 | 0.999 | 0.791 |
| *13* | *Acetophenone* | 0.176 | 0.034 | 0.167 | 0.123 | 0.290 | 0.781 | 0.402 | 0.914 |
| *14* | *o-Tolunitrile* | 0.303 | 0.047 | 0.341 | 0.141 | 0.422 | 0.757 | 0.606 | 0.904 |
| *15* | *m-Tolunitrile* | 0.106 | 0.029 | 0.195 | 0.079 | 0.120 | 0.373 | 0.543 | 0.946 |
| *16* | *p-Tolunitrile* | 0.138 | 0.022 | 0.321 | 0.149 | 0.093 | 0.510 | 0.375 | 0.971 |
| *17* | *Benzyl nitrile* | 0.056 | 0.012 | 0.368 | 0.134 | 0.063 | 0.247 | 0.314 | 1.000 |
| *18* | *Naphthalene* | 0.341 | 0.123 | 0.332 | 0.192 | 0.132 | 0.319 | 0.470 | 1.000 |
| *19* | *Benzo[b]thiophene* | 0.382 | 0.116 | 0.426 | 0.263 | 0.207 | 0.414 | 0.501 | 1.000 |
| *20* | *Benzothiazole* | 0.049 | 0.015 | 0.038 | 0.117 | 0.011 | 0.331 | 0.083 | 1.000 |
| *21* | *Quinoline* | 0.037 | 0.050 | 0.031 | 0.093 | 0.018 | 0.206 | 0.095 | 1.000 |
| *22* | *Isoquinoline* | 0.051 | 0.012 | 0.007 | 0.241 | 0.000 | 0.061 | 0.000 | 1.000 |
| *23* | *1,3-Dicyanobenzene* | 0.022 | 0.006 | 0.038 | 0.300 | 0.000 | 0.143 | 0.037 | 1.000 |
| *24* | *Indole* | 0.034 | 0.017 | 0.124 | 0.365 | 0.017 | 0.141 | 0.154 | 1.000 |
| *25* | *2-Methylnaphthalene* | 0.207 | 0.116 | 0.207 | 0.194 | 0.024 | 0.151 | 0.261 | 1.000 |
| *26* | *1-Methylnaphthalene* | 0.247 | 0.156 | 0.222 | 0.223 | 0.042 | 0.206 | 0.297 | 1.000 |
| *27* | *1,2-Dicyanobenzene* | 0.017 | 0.014 | 0.028 | 0.236 | 0.000 | 0.119 | 0.026 | 1.000 |
| *28* | *Biphenyl* | 0.129 | 0.160 | 0.117 | 0.222 | 0.007 | 0.111 | 0.138 | 1.000 |
| *29* | *2-Ethylnaphthalene* | 0.086 | 0.088 | 0.118 | 0.206 | 0.000 | 0.039 | 0.140 | 1.000 |
| *30* | *2,6-Dimethylnaphthalene* | 0.070 | 0.089 | 0.101 | 0.265 | 0.000 | 0.056 | 0.135 | 1.000 |
| *31* | *1,4-Dimethylnaphthalene* | 0.053 | 0.083 | 0.058 | 0.139 | 0.000 | 0.041 | 0.032 | 1.000 |
| *32* | *Acenaphtylene* | 0.128 | 0.157 | 0.134 | 0.303 | 0.004 | 0.082 | 0.149 | 1.000 |
| *33* | *Acenaphthene* | 0.090 | 0.185 | 0.188 | 0.439 | 0.003 | 0.048 | 0.110 | 0.801 |
| *34* | *1-Naphthalenecarbonitrile* | 0.055 | 0.147 | 0.069 | 0.310 | 0.002 | 0.059 | 0.089 | 1.000 |
| *35* | *4-Methylbiphenyl* | 0.029 | 0.168 | 0.052 | 0.357 | 0.000 | 0.019 | 0.062 | 1.000 |
| *36* | *2-Naphthalenecarbonitrile* | 0.022 | 0.093 | 0.039 | 0.362 | 0.000 | 0.020 | 0.052 | 1.000 |
| *37* | *Fluorene* | 0.069 | 0.230 | 0.074 | 0.344 | 0.000 | 0.032 | 0.095 | 1.000 |
| *38* | *Diphenylamine* | 0.039 | 0.213 | 0.064 | 0.659 | 0.000 | 0.051 | 0.058 | 1.000 |
| *39* | *Benzophenone* | 0.003 | 0.084 | 0.346 | 1.000 | 0.000 | 0.009 | 0.000 | 0.403 |
| *40* | *Phenanthrene* | 0.022 | 0.447 | 0.039 | 0.544 | 0.000 | 0.010 | 0.051 | 1.000 |
| *41* | *Anthracene* | 0.002 | 0.241 | 0.042 | 0.673 | 0.000 | 0.004 | 0.000 | 0.961 |
| *42* | *Carbazole* | 0.000 | 0.208 | 0.042 | 0.848 | 0.000 | 0.005 | 0.021 | 0.904 |
| *43* | *Ethyl centralite* | 0.157 | 0.888 | 0.119 | 0.486 | 0.003 | 0.176 | 0.109 | 0.750 |
| *44* | *2-Nitrodiphenylamine* | 0.000 | 0.234 | 0.000 | 0.986 | 0.000 | 0.000 | 0.000 | 0.688 |
| *45* | *Dibutyl phthalate* | 0.066 | 0.554 | 0.260 | 0.780 | 0.000 | 0.010 | 0.194 | 0.729 |
| *46* | *Methyldiphenylurea* | 0.000 | 0.000 | 0.000 | 1.000 | 0.000 | 0.000 | 0.000 | 0.000 |
| *47* | *Fluoranthene* | 0.002 | 0.654 | 0.036 | 0.686 | 0.000 | 0.004 | 0.015 | 0.829 |
| *48* | *Pyrene* | 0.011 | 0.740 | 0.048 | 0.712 | 0.000 | 0.003 | 0.065 | 0.780 |
| *49* | *4-Nitrodiphenylamine* | 0.000 | 0.500 | 0.000 | 0.205 | 0.000 | 0.000 | 0.000 | 0.500 |
| *50* | *Chrysene* | 0.000 | 0.640 | 0.000 | 0.963 | 0.000 | 0.000 | 0.000 | 1.000 |
|  |  |  |  |  |  |  |  |  |  |
|  | *MEAN* | 0.116 | 0.156 | 0.131 | 0.315 | 0.180 | 0.295 | 0.302 | 0.775 |
|  | *MEDIAN* | 0.068 | 0.084 | 0.083 | 0.222 | 0.005 | 0.142 | 0.139 | 0.953 |

Performance ranking for each target analyte


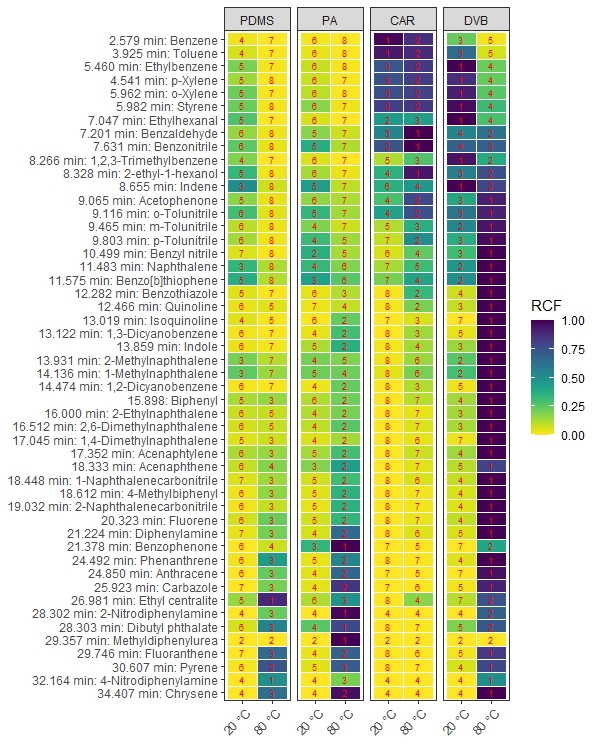


**Figure S3** – Heatmap comparing the between-ammunition relative concentration coefficients (RCFs) observed at any condition tested in this work for each target analyte (*n* = 50). The numbers in the cells are the ranks. Times in the labels are the retention times (*t_R_*).

Between-ammunition CVs by analyte

**Table S6** – Between ammunition coefficients of variation (CVs) observed at each extraction condition. These were obtained by averaging the CVs determined after the extraction of each ammunition type (*n* = 4).

| ***Compound*** | | ***Between-ammunition CV [%]*** | | | | | | | |
| --- | --- | --- | --- | --- | --- | --- | --- | --- | --- |
|  |  | ***PDMS*** | | ***PA*** | | ***CAR*** | | ***DVB*** | |
|  |  | ***@20°C*** | ***@80°C*** | ***@20°C*** | ***@80°C*** | ***@20°C*** | ***@80°C*** | ***@20°C*** | ***@80°C*** |
| *1* | *Benzene* | 17.6 | 31.6 | 15.7 | 10.0 | 7.5 | 22.1 | 25.6 | 22.2 |
| *2* | *Toluene* | 18.3 | 29.4 | 16.5 | 10.1 | 9.7 | 21.5 | 25.9 | 21.0 |
| *3* | *Ethylbenzene* | 19.2 | 99.8 | 17.6 | 41.7 | 11.8 | 16.1 | 25.7 | 20.1 |
| *4* | *p-Xylene* | 15.9 | 48.2 | 14.3 | 49.7 | 18.0 | 15.4 | 21.0 | 18.4 |
| *5* | *o-Xylene* | 17.7 | 81.8 | 18.4 | 75.2 | 25.8 | 18.8 | 20.1 | 16.7 |
| *6* | *Styrene* | 23.6 | 26.5 | 20.5 | 15.3 | 19.5 | 19.4 | 24.1 | 16.6 |
| *7* | *2-Ethylhexanal* | 20.3 | N/A | 86.8 | N/A | 16.0 | 19.5 | 42.8 | 16.2 |
| *8* | *Benzaldehyde* | 40.9 | 46.6 | 32.6 | 20.4 | 18.1 | 23.4 | 16.4 | 24.0 |
| *9* | *Benzonitrile* | 28.9 | 33.1 | 21.4 | 9.9 | 19.2 | 23.4 | 16.5 | 21.2 |
| *10* | *1,2,3-Trimethylbenzene* | 42.7 | N/A | 17.1 | N/A | 26.0 | 86.6 | 30.4 | 44.8 |
| *11* | *2-Ethyl-1-hexanol* | 24.8 | 26.9 | 18.0 | 17.8 | 39.1 | 21.7 | 16.5 | 22.6 |
| *12* | *Indene* | 19.7 | 27.3 | 21.7 | 16.6 | 13.7 | 16.6 | 19.2 | 10.6 |
| *13* | *Acetophenone* | 49.4 | 58.6 | 37.1 | 19.3 | 21.5 | 24.8 | 22.4 | 31.0 |
| *14* | *o-Tolunitrile* | 26.5 | 70.5 | 24.2 | 9.1 | 24.1 | 18.6 | 19.8 | 22.2 |
| *15* | *m-Tolunitrile* | 77.7 | 41.0 | 24.5 | 9.0 | 69.5 | 15.8 | 9.8 | 29.5 |
| *16* | *p-Tolunitrile* | 65.5 | 88.4 | 33.2 | 8.1 | 85.3 | 23.5 | 62.3 | 29.1 |
| *17* | *Benzyl nitrile* | 90.9 | 45.4 | 58.7 | 16.0 | 22.9 | 42.5 | 44.0 | 43.7 |
| *18* | *Naphthalene* | 22.1 | 27.9 | 25.5 | 12.4 | 21.5 | 15.1 | 14.9 | 28.1 |
| *19* | *Benzo[b]thiophene* | 33.8 | 23.2 | 36.9 | 22.4 | 33.5 | 20.2 | 14.0 | 30.9 |
| *20* | *Benzothiazole* | 27.5 | 141.4 | 11.4 | 30.8 | 141.4 | 63.0 | 78.8 | 29.3 |
| *21* | *Quinoline* | 76.5 | 47.5 | 60.3 | 22.6 | 9.8 | 20.9 | 49.2 | 19.6 |
| *22* | *Isoquinoline* | 20.2 | 141.4 | 141.4 | 3.8 | N/A | 78.8 | N/A | 56.5 |
| *23* | *1,3-Dicyanobenzene* | 16.1 | 82.1 | 35.4 | 56.7 | N/A | 38.6 | 8.6 | 69.0 |
| *24* | *Indole* | 33.3 | 67.7 | 35.4 | 34.2 | 28.2 | 76.3 | 25.8 | 82.2 |
| *25* | *2-Methylnaphthalene* | 24.3 | 36.0 | 30.1 | 12.5 | 79.4 | 13.8 | 16.3 | 41.1 |
| *26* | *1-Methylnaphthalene* | 25.0 | 39.4 | 27.7 | 15.3 | 63.1 | 12.4 | 18.7 | 38.7 |
| *27* | *1,2-Dicyanobenzene* | 11.2 | 101.7 | 53.5 | 43.2 | N/A | 41.4 | 10.1 | 90.1 |
| *28* | *Biphenyl* | 37.7 | 51.9 | 36.1 | 20.0 | 35.1 | 22.7 | 21.1 | 45.9 |
| *29* | *2-Ethylnaphthalene* | 40.5 | 110.0 | 14.7 | 13.1 | N/A | 10.8 | 55.4 | 46.2 |
| *30* | *2,6-Dimethylnaphthalene* | 40.6 | 114.5 | 14.6 | 30.2 | N/A | 10.7 | 52.8 | 48.7 |
| *31* | *1,4-Dimethylnaphthalene* | 87.3 | 100.5 | 81.1 | 101.1 | N/A | 5.3 | 29.1 | 41.6 |
| *32* | *Acenaphtylene* | 31.6 | 43.7 | 32.8 | 28.1 | 20.3 | 11.7 | 17.8 | 39.0 |
| *33* | *Acenaphthene* | 17.61 | 31.6 | 15.7 | 10.0 | 7.5 | 22.1 | 25.6 | 22.2 |
| *34* | *1-Naphthalenecarbonitrile* | 28.9 | 44.7 | 65.4 | 15.8 | 10.1 | 13.9 | 21.5 | 45.6 |
| *35* | *4-Methylbiphenyl* | 49.6 | 54.1 | 29.8 | 26.0 | 19.0 | 17.8 | 26.4 | 36.4 |
| *36* | *2-Naphthalenecarbonitrile* | 80.7 | 58.9 | 96.5 | 12.7 | N/A | 15.5 | 77.5 | 30.7 |
| *37* | *Fluorene* | 78.9 | 63.6 | 100.4 | 22.5 | N/A | 40.8 | 62.5 | 43.7 |
| *38* | *Diphenylamine* | 37.1 | 44.1 | 33.6 | 18.7 | 141.4 | 11.8 | 20.7 | 42.1 |
| *39* | *Benzophenone* | 43.4 | 29.9 | 20.1 | 23.3 | 28.7 | 31.7 | 12.8 | 21.1 |
| *40* | *Phenanthrene* | 141.4 | 74.0 | 35.3 | 22.2 | N/A | 74.3 | N/A | 56.6 |
| *41* | *Anthracene* | 56.7 | 49.3 | 30.3 | 25.5 | N/A | 30.9 | 23.1 | 42.9 |
| *42* | *Carbazole* | 141.4 | 72.7 | 95.4 | 20.8 | N/A | 141.4 | N/A | 54.8 |
| *43* | *Ethyl centralite* | N/A | 79.7 | 41.3 | 40.7 | N/A | 74.1 | 77.9 | 38.9 |
| *44* | *2-Nitrodiphenylamine* | 56.0 | 50.6 | 30.4 | 47.6 | 46.1 | 63.1 | 28.2 | 49.2 |
| *45* | *Dibutyl phthalate* | N/A | 123.8 | N/A | 78.9 | N/A | N/A | N/A | 89.5 |
| *46* | *Methyldiphenylurea* | 46.4 | 71.3 | 48.2 | 45.5 | N/A | 100.7 | 34.6 | 49.1 |
| *47* | *Fluoranthene* | N/A | N/A | N/A | 89.9 | N/A | N/A | N/A | N/A |
| *48* | *Pyrene* | 70.7 | 53.8 | 66.5 | 42.3 | N/A | 72.6 | 84.7 | 52.5 |
| *49* | *4-Nitrodiphenylamine* | 99.8 | 54.7 | 22.1 | 40.1 | N/A | 72.4 | 32.7 | 49.8 |
| *50* | *Chrysene* | N/A | 141.4 | N/A | 80.7 | N/A | N/A | N/A | 141.4 |
|  |  |  |  |  |  |  |  |  |  |
|  | *MEAN* | 45.7 | 65.8 | 39.8 | 32.7 | 36.3 | 35.5 | 31.6 | 42.9 |
|  | *MEDIAN* | 37.1 | 54.1 | 31.5 | 22.4 | 22.9 | 21.9 | 24.1 | 39.0 |
